# Genes Involved in the Balance between Neuronal Survival and Death during Inflammation

**DOI:** 10.1371/journal.pone.0000310

**Published:** 2007-03-21

**Authors:** Isaias Glezer, Ariel Chernomoretz, Samuel David, Marie-Michèle Plante, Serge Rivest

**Affiliations:** 1 Laboratory of Molecular Endocrinology, Centre Hospitalier de l'Université Laval (CHUL) Research Center and Department of Anatomy and Physiology, Laval University, Laurier, Québec, Canada; 2 Physics Department, FCEyN, University of Buenos Aires, Buenos Aires, Argentina; 3 Center for Research in Neuroscience, Research Institute of the McGill University Health Center, Montreal General Hospital Research Institute, Montreal, Quebec, Canada; Free University of Brussels, Belgium

## Abstract

Glucocorticoids are potent regulators of the innate immune response, and alteration in this inhibitory feedback has detrimental consequences for the neural tissue. This study profiled and investigated functionally candidate genes mediating this switch between cell survival and death during an acute inflammatory reaction subsequent to the absence of glucocorticoid signaling. Oligonucleotide microarray analysis revealed that following lipopolysaccharide (LPS) intracerebral administration at striatum level, more modulated genes presented transcription impairment than exacerbation upon glucocorticoid receptor blockage. Among impaired genes we identified ceruloplasmin (Cp), which plays a key role in iron metabolism and is implicated in a neurodegenative disease. Microglial and endothelial induction of Cp is a natural neuroprotective mechanism during inflammation, because Cp-deficient mice exhibited increased iron accumulation and demyelination when exposed to LPS and neurovascular reactivity to pneumococcal meningitis. This study has identified genes that can play a critical role in programming the innate immune response, helping to clarify the mechanisms leading to protection or damage during inflammatory conditions in the CNS.

## Introduction

The presence of infection is recognized by receptors for specific elements called the pathogen-associated molecular patterns (PAMPs) that are produced by microorganisms. Recognition of these PAMPs by antigen-presenting cells is the first step of a complex inflammatory reaction that characterizes the innate immune response [1]. Lipopolysaccharide (LPS), a major component of the outer membranes of Gram-negative bacteria, is the best-known target of innate recognition and induces a robust inflammatory response by antigen-presenting cells. The endotoxin activates a transient innate immune reaction via Toll-like receptor 4 (TLR4) and the rapid interaction of its Toll/IL-1R homology domain with myeloid differentiation factor 88 [2], [3]. This leads to downstream signaling that activates NF-κB, a critical player in transcriptional activation of numerous pro-inflammatory genes [4]. Interaction of LPS with its cognate TLR4 can also activate interferon pathway through other adaptor and signaling molecules [1]. Collectively, these signaling events and transcriptional processes are essential for eliminating pathogens and preparing the transfer to a more specific acquired immune response.

Glucocorticoids (GCs) are potent endogenous anti-inflammatory molecules. Activated GC receptors (GRs) are able to interfere with the transactivation potential of the p65 NF-κB subunit as well as AP-1. Other mechanisms explaining the effects of GCs on gene transcription have been proposed, including the competition between GRs and NF-κB for nuclear coactivators, such as CREB-binding protein and p300 (CBP/p300) [5]. The mechanisms involved in the effects of GCs on gene expression in the central nervous system (CNS) remains largely unknown [6].

Intracerebral LPS administration causes a strong and time-dependent transcriptional activation of inflammatory genes in microglial cells ipsilateral to the site of injection and this acute reaction is not associated with neuronal damage or demyelination [7], [8]. However, the inflammatory response lasts longer in the brain of animals that are treated with the GR antagonist RU486 before the endotoxin, which then becomes highly toxic to neural cells [9], [10]. These data provide evidence that without a proper inhibitory feedback by GCs, an acute inflammatory response may have serious detrimental consequences for the brain.

Here we aimed to determine the genes that are differentially regulated by LPS in the presence or absence of the GR signaling. We took advantage of this unique *in vivo* model to identify the key players involved in the delicate balance between cell survival and death during a natural innate immune reaction in the CNS. While the use of neurotoxins is limited to access the contribution of the innate immunity to brain damage due to either direct or indirect microglia activation by their ability to cause neuronal death, the model used in the present study is unique and appropriated in this regard. The comparison between LPS alone and LPS combined to RU486 offers an adequate paradigm because the deregulated innate immune reaction is directly responsible for the cell fate. Functional analysis of the differentially regulated genes between the two conditions led to the selection of ceruloplasmin (Cp) as a candidate protective gene and to the investigation of its role in neuroinflammatory contexts, providing a model to illustrate the beneficial mechanisms engaged by the CNS innate immune response.

## Results

### Functional analysis of the differential neuroinflammatory gene expression in presence or absence of endogenous GCs

Our design allowed the detection of 345 probe sets representing 286 different genes (gene IDs) significantly modulated 12 h after intrastriatal LPS injection in the ipsilateral side of the injection (no significant changes were observed in contralateral side). These probes are listed in the dataset S1. The 12 h time-point was selected to avoid early gene expression noise caused by mechanical damage and late confounding patterns originating from potential damage subsequent to the LPS/RU486 treatment. Approximately 86.3% of the genes modulated by LPS were upregulated while ∼14.7% were downregulated. Among the upregulated genes, many were linked to the inflammatory signaling. In addition to these expected data, genes induced by LPS in CNS are linked to various biological processes that range from acute-phase response to cell differentiation and angiogenesis. Downregulated genes by the endotoxin included mainly transcription factors and those encoding proteins related to brain development, metabolism, myelination and transport. This information can be found in figure 2, dataset S1 and figure S1.

**Figure 1:**
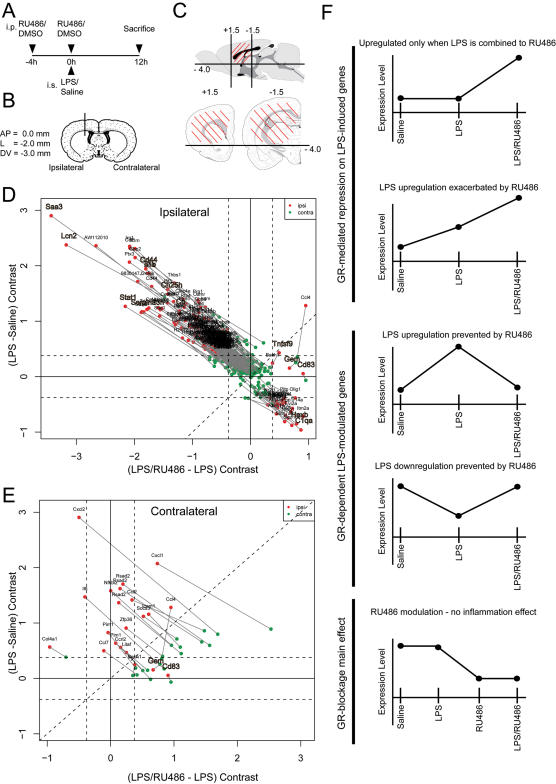
Gene profiling of CNS LPS response in presence or absence of functional GR signaling. (A) Treatment schedule of animals. (B and C) Schematic representation of the injection site and brain area dissected for RNA isolation, respectively. (D) A 2-contrast plot of genes selected as differently expressed in ipsilateral side when LPS injection was performed in mice pre-treated or not with RU486. The abscissa axis represents the difference (contrast) between LPS/RU486 treatment and LPS single treatment (RMA expression levels); ordinate axis represents the LPS *vs*. Saline contrast. Red dots represent ipsilateral contrast value; green dots, represent contralateral contrast values. Genes highlighted in the diagram were selected for validation experiments. Dashed lines: 30% fold-change. (E) Similar to “D”, but in regard to contralateral side. (F) Representation of the gene expression behavior associated with RU486 effects on LPS-induced changes, defining how these groups of genes are described in the text. Abbreviations: AP, antero-posterior; contra, contralateral; DV, dorso-ventral; i.p., intra-peritoneal; ipsi, ipsilateral; i.s., intrastriatal; L, lateral.

**Figure 2:**
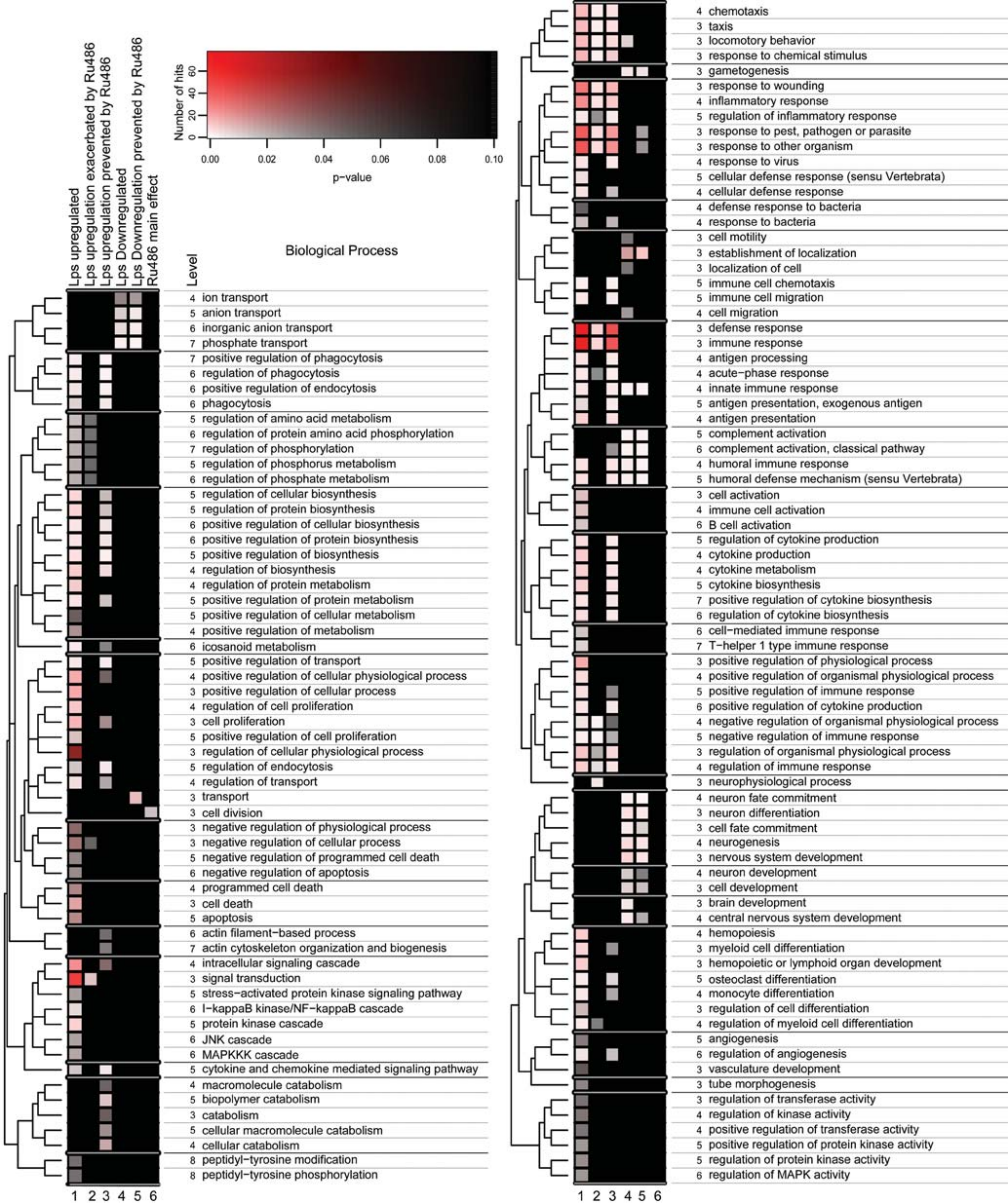
Functional classification and biological relevance of the differently expressed genes. The plot showing significant biological processes (hypergeometric distribution) associated with six different lists of differently expressed genes (LPS upregulated, LPS upregulation exacerbated by RU486, LPS upregulation prevented by RU486, LPS downregulated, LPS dowregulation prevented by RU486 and RU486 main effect). Hierarchical clustering of the Gene Ontology nodes was performed as described in text S1. A color/intensity code assigns number of genes and p-value for each biological process associated with the lists' heat map.

Regarding gene expression comparing LPS effects in presence or absence of RU486, 235 probe sets (representing 200 different gene IDs) were selected according to our strategy (listed in dataset S2). It should be noted that these probe sets do not overlap with genes that are modulated by RU486 independently of LPS and/or side effect interactions (GR-blockage main effect; 47 probe sets-see figure S1 C). As depicted by the figure 1D (ipsilateral side), most genes differently expressed when evaluating the effect of RU486 on LPS-treatment (LPS/RU486–LPS contrast) were those wherein LPS upregulation was prevented (or partially prevented in at least 50%) by GR blockage. This gene subset represents approximately 54.9% of the LPS modulated genes (upregulated and downregulated). In general, genes with such behavior were found across many categories of biological processes (Fig. 2). Unexpectedly, genes related to host response against pathogens and remarkably, viral and interferon response, were also upregulated by LPS in a manner that is prevented by RU486 (see also figure S1 A, B).

In contrast, only 18 genes presented a GR-mediated repression on LPS-induced gene expression, many of them detected as differently expressed in the contralateral side (Fig. 1E). These genes can be divided into: a) those already upregulated by LPS, such as chemokines, cytokines, JAK-STAT pathway, regulators of transcription and others; b) genes only induced when LPS is combined to RU486, namely a cell-surface antigen of activated dendritic cell (Cd83), a GTPase of the RGK family (Gem) and a transcription factor of the CNC-bZip family (Bach1).

Similarly to the transcripts that were upregulated following LPS administration, RU486 prevented the dowregulation of 25 out of 42 genes, implying that endogenous GCs are required for both upregulation and downregulation of a considerable number of genes. A comprehensive exploration of microarray data is illustrated by the figure S1 (A–F), while figure 2 shows the main findings related to biological process according to Gene Ontology (GO) classification.

### Validation and anatomical distribution of representative genes modulated by GR and LPS signaling in the CNS

**Figure 3:**
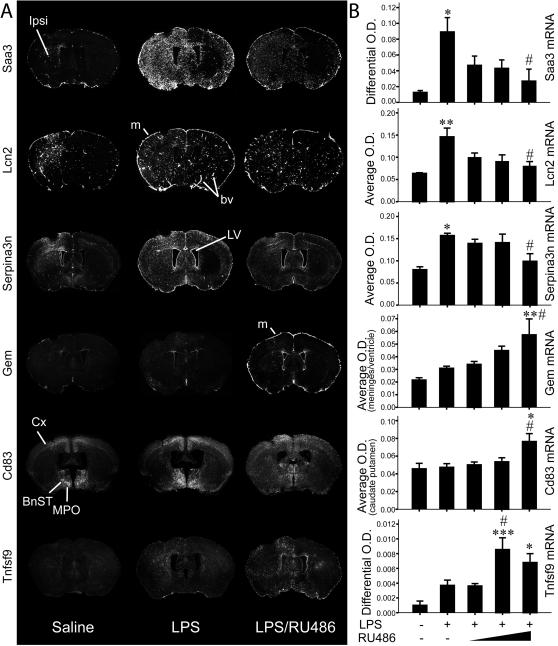
Bimodal RU486 effect on CNS inflammation as demonstrated by APRs and genes only responsive to LPS upon GR-antagonism. (A) Dark-field photomicrographs showing hybridization signals of the specified transcripts from mice that received saline or, LPS or, LPS/RU486 combined treatments. (B) Semi-quantitative analysis of mRNA levels [optical density (O.D.)] in mouse brains 12 h after the intracerebral infusions. Absence (-) or presence (+) of LPS or increasing doses of RU486 is indicated. Quantification methods may vary among transcripts; see “Experimental Procedures” section. Results represent means ± SEM of 3–5 mice per group. One-way ANOVA followed by a Tukey's HSD multiple comparison test: significantly different (* p<0.05, ** p<0.01, *** p<0.005) from the saline-injected group; significantly different (# p<0.05) from the LPS-treated group. Abbreviations: BnST, bed nucleus of stria terminalis; bv, blood vessel; Cx, cortex; Ipsi, ipsilateral; LV, lateral ventricle; m, meninges; MPO, medial preoptic nucleus.

Several genes were selected for validation by *in situ* hybridization according to transcript behavior and functional relevance. These included acute-phase reactants (APRs) (Saa3, Lcn2 and Serpina3n), genes with GR-mediated repression on LPS induction (Gem, Cd83 and Tnfsf9) and other transcripts modulated by GR signaling. In this particular case, RU486 essentially prevented gene upregulation (Cd44, Stat1, Ch25h and Il1b) or downregulation (C1qa and Hexb) by LPS. Figure 3A shows representative examples of Saa3, Lcn2 (N-GAL/24p3), Serpina3n, Gem, Cd83 and Tnfsf9 (4-1BBL) transcripts in the dorsal basal ganglia of mice that received saline injection, LPS or LPS/RU486; wherein the different pattern of expression is quite evident among transcripts. Interestingly, Gem expression localized over cells lining the meninges, while Cd83 and Tnfsf9 transcripts were distributed across parenchymal elements of the brain. Moreover, Cd83 shows convincing constitutive neuronal expression (Fig. 3A). Figure 3B illustrates the dose-dependent effect of RU486 on brain reaction to LPS.

**Figure 4:**
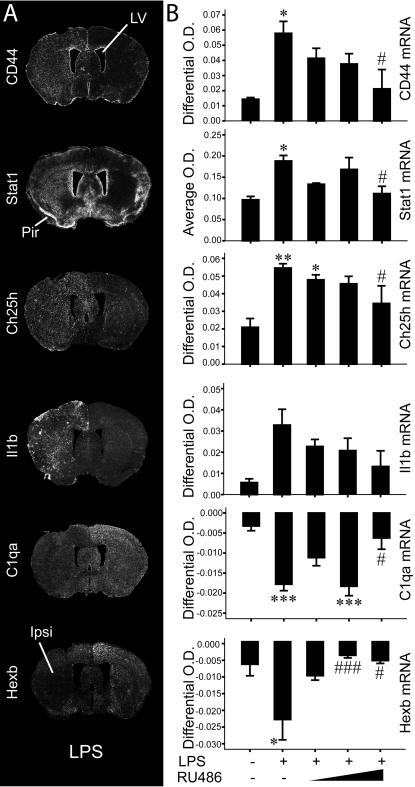
Validation of genes upregulated or downregulated by LPS in a glucocorticoid-dependent manner. (A) Representative examples of coronal sections depicting LPS induced changes in gene expression of the indicated transcripts. (B) Semi-quantitative analysis of mRNA levels (O.D.) in mouse brains 12 h after the intracerebral infusions. One-way ANOVA followed by a Tukey's HSD multiple comparison test: significantly different (* p<0.05, ** p<0.01, *** p<0.005) from the saline-injected group; significantly different (# p<0.05, ### p<0.005) from the LPS-treated group. Abbreviations: Ipsi, ipsilateral; LV, lateral ventricle; Pir, piriform cortex.

Figure 4 depicts results for the validation of Cd44, Stat1, Ch25h, Il1b, C1qa and Hexb. The anatomical distribution of Cd44 (probed for the five first exons) and Ch25h was quite identical, but very different from Stat1 (neuronal-like) and Il1b. Unlike Cd44 and Ch25h, Stat1 did not seem to response to RU486 in a dose-dependent manner and Il1b gene expression was not significantly different among groups (ANOVA). For the latter transcript, a significant linear trend between induced mRNA levels and increasing doses of RU486 was reached (p<0.05; trend analysis). Noteworthy, the cRNA probe used was designed to hybridize the same target region present in affymetrix gene chips.

The expression pattern of C1qa and Hexb is highly suggestive of ubiquitous constitutive expression, which was selectively repressed by the endotoxin in the ipsilateral side of injection (Fig. 4B). Because LPS-gene profiling indicated repression of the complement classical pathway [C1qa, C1qb and C1qc downregulation and Serping1 (C1-Inhibitor) upregulation], we further characterized the temporal dynamic of this process (Figure S2).

According to our double labeling procedure, Cd44, Tnfsf9 and Cd83 are expressed mainly by microglial cells, Lcn2 by endothelial cells and Saa3 transcript by microglia and few astrocytes. In contrast, Stat1 is mainly produced by neurons (Figure S3).

### The cellular characterization of Cp: a candidate gene dependent on GR and LPS signaling

As our selection of candidate genes relied on a potential protective role, we screened genes based on their natural regulation in presence of an intact LPS and GR signaling. As depicted by the figure 1D, many genes had the desired behavior and we looked for those that had been previously linked to neurodegenerative disorders. We found ceruloplasmin (Cp) that was strongly induced by LPS (neuroprotection) and completely inhibited by the GR blockade (a neurotoxic condition). *Cp* gene encodes a protein with ferroxidase activity and loss of function mutations in this gene leads to accumulation of iron and human neurodegenerative disease (see discussion). We then postulated that induction of Cp might be a potent neuroprotective mechanism during acute inflammation.

**Figure 5:**
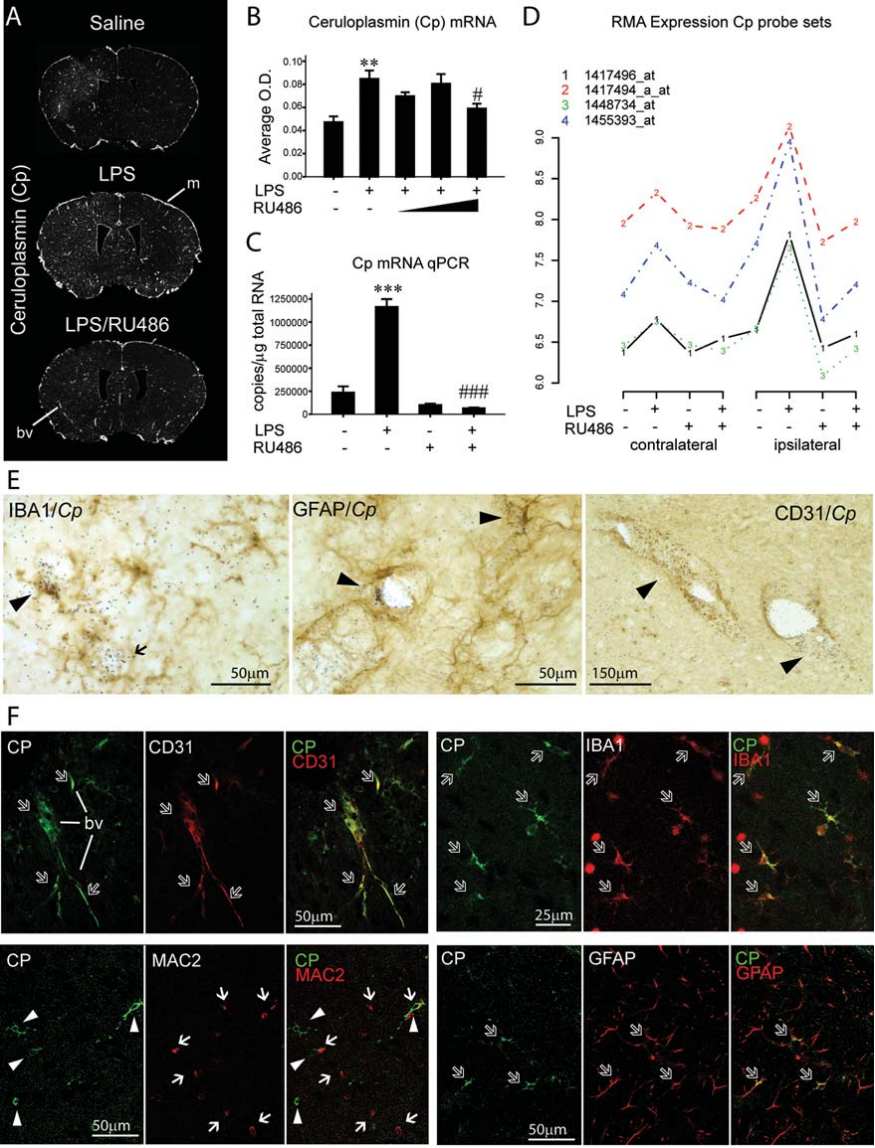
Characterization of ceruloplasmin (Cp) expression following an acute innate immune challenge. (A) Hybridization signals of Cp riboprobe on brain sections of saline-, or LPS-, or LPS/RU486-treated mice, sacrificed 12 h after the intrastriatal injection. (B) Semi-quantitative analysis of Cp mRNA levels (O.D.) depicted in “A”. (C) Results of quantitative reverse-transcriptase PCR (qPCR). (D) RMA expression levels of four different probe sets to detect Cp transcript selected according to a significant LPS effect. (E) Representative microphotographs of labeling experiments combining immunohistochemistry with *in situ* hybridization. Silver grain signals (*Cp mRNA*) overlap IBA1-, GFAP- and CD31-positive cells. (F) Confocal microscopy results showing co-localization of CP protein with CD31-, IBA1- and GFAP-positive cells, but not with MAC-2-positive cells, in brain sections from animals sacrificed 24 h after LPS challenge. Dark arrowheads: co-localization of silver grains with immunolabeling brown staining. Empty white arrows: co-localization determined by confocal laser scanning. Full white arrows and white arrowheads were used to identify cell types that failed to show convincing co-localization. One-way ANOVA followed by a Tukey'S HSD multiple comparison test: significantly different (** p<0.01, *** p<0.005) from the saline-injected group; significantly different (# p<0.05, ### p<0.005) from the LPS-treated group. Abbreviations: bv, blood vessel; m, meningis.

**Figure 6:**
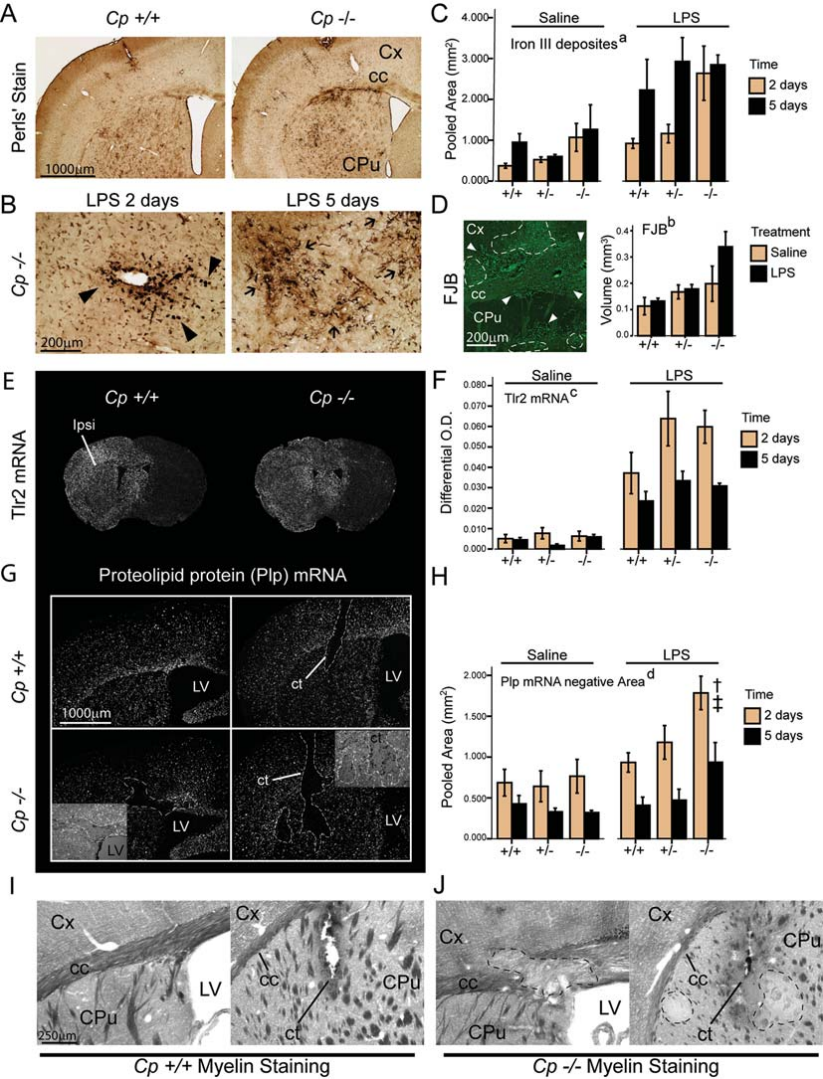
Ceruloplasmin regulates brain iron levels during inflammation and confers neuroprotection. (A) *Cp*+/+, +/- or -/- mice were sacrificed 2 days after LPS infusion and brain sections stained for Iron III (Perls' staining). (B) High magnification of iron labeling (representative brain sections from *Cp*-/-mice sacrificed 2 or 5 days after LPS infusion). (C) Surface analysis to determine extent of iron deposition. (D) Fluoro-Jade B (FJB) staining was performed in brain sections from *Cp*+/+, +/- and -/- infused with LPS or saline. Volumetric analysis was performed by stereological procedure. (E) Representative dark-field picture of adjacent brain sections hybridized with Tlr2 cRNA probe in LPS treated mice 2 days after infusion. (F) Semi-quantitative analysis of Tlr2 mRNA hybridization signal. (G) Representative sections hybridized with Plp cRNA riboprobe to detect regions lacking signals (demyelinated area). These panels show two different coronal levels (cannulae track level and a section distal to the site of injection). (H) Surface analysis of demyelinated area. (I) and (J) are representative microphotographs of Sudam Black B (SBB) stained sections from *Cp*+/+ and -/- mice sacrificed 2 days after LPS infusion, as indicated. Statistical analysis was performed via a two or three-way ANOVA followed by a Bonferroni's multiple comparison test when applicable. “a”, significant genotype effect (p<0.05), significant time effect (p<0.01), significant treatment effect (p<0.001), *Cp*-/-significantly different from *Cp*+/+ (p<0.01) and *Cp*+/- (p<0.05). “b”, significant genotype effect (p<0.01), *Cp*-/- statistically different from *Cp*+/+ (p<0.005). “c”, significant interaction between treatment and time factors (p<0.01), LPS treatment 2 days statistically different from LPS 5 days (p<0.001). “d”, significant interaction between treatment and genotype effect (p<0.05), significant time effect (p<0.001), in the LPS treated group *Cp*-/- was statistically different from *Cp*+/+ († p<0.005) and from *Cp*+/- (‡ p<0.01). Abbreviations: cc, corpus callosum; CPu, caudate putamen; ct, cannulae track; Cx, cortex; LV, lateral ventricle.

The regulation of that transcript was quantified by different complementary approaches. Quantification of *in situ* hybridization data indicated that RU486 significantly prevented LPS-induced Cp gene expression, but only at the higher dose tested (Fig. 5A, B). Nevertheless, this effect of RU486 was confirmed with a lower dose by quantitative RT-PCR (Fig. 5C). In addition, figure 6D shows that the four different probe sets used in the gene chip generated the same data, which all together provide very solid evidence that GR blockage is able to prevent LPS-induced Cp gene expression. We then determined the type of cells that synthesize this protein during an acute immune challenge and found co-localization of Cp mRNA with IBA1-, GFAP- and CD31-immunoreactive (ir) cells (Fig. 5E). Confocal laser microscopy was also used to accurately distinguish the different types of cells with positive Cp-ir signal. As depicted by the figure 5F, Cp protein strongly co-localized with endothelial cells and IBA1-postive microglia and co-localization with GFAP-astrocytes was rare. MAC-2 is frequently used as a marker of a subtype of activated microglia, but it failed to express Cp (Fig. 5F). It is worth mentioning that the shape of MAC-2-positive cells was quite different from that of IBA1-positive microglia (Figure S4), suggesting the possibility that Cp is produced by the resident ramified microglia (IBA1) and not amoeboid-like or infiltrating myeloid cells (MAC-2).

A robust extracellular CP-ir signal was detected in the CNS of LPS-injected mice at time-point 2 days post injection. Such a staining largely declined 3 days later suggesting a rapid and transient secretion of this protein during inflammation. As expected, *Cp*-/-mice failed to exhibit any CP-ir signal following similar treatments (Figure S5).

### Neuroprotective role of Cp during an acute inflammatory reaction

Due to the involvement of Cp in iron metabolism, we measured iron accumulation in the mouse brain following a single intracerebral LPS bolus. As depicted by the figure 6A, B and C, LPS was able to increase iron III deposition in the ipsilateral side of injection in both *Cp*+/+ and *Cp*-/-mice. Interestingly, the cells containing iron III had a different morphology over time, being frequently round at 2 days post-injection and changing to a ramified shape at 5 days (Fig. 6B). *Cp*-deficient mice had a dramatic increase in iron III deposits following LPS administration, especially at time-point 2 days. Accentuated iron accumulation in *Cp*-/-animals seems to correlate with neurodegeneration in this genotype, at least as evaluated by the fluorescent marker Fluoro Jade B (FJB, Fig. 6D).

The gene encoding Tlr2 is induced in microglia during innate immunity, but no significant change was found between both groups of mice treated with the endotoxin (Fig. 6E, F), indicating that microglia reactivity to LPS is Cp independent. Thereafter, we evaluated whether Cp may play a role in protecting the CNS white matter from an acute inflammatory challenge. Surface analysis of Proteolipid protein 1 (Plp1) mRNA levels rapidly decrease during demyelination or myelin-associated cell death [11]. Figure 6 (panel G and H) shows representative examples of such demyelinating processes in *Cp*-/-and not in *Cp*+/+or *Cp*+/-mice. These data were corroborated by the myelin SBB staining (Fig. 6I, J), which all together provide evidence that the endotoxin can indeed provoke demyelination, but only in mice that bear a mutation in the *Cp* gene.

### Role of Cp in Streptococcus pneumoniae-induced meningitis

**Figure 7:**
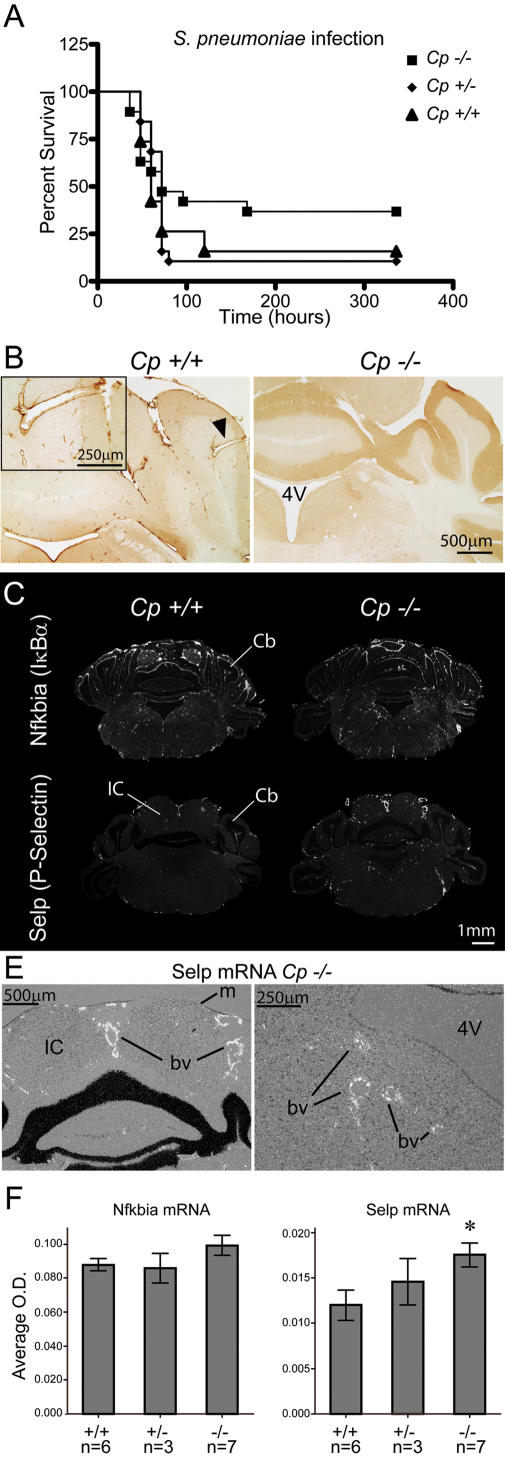
Modulation of cerebro-vascular response by ceruloplasmin during early phases of pneumococcal infection. (A) Survival curves of *Cp*+/+, +/- and -/- following inoculation with *S. Penumoniae* (2 week follow up). (B) Ceruloplasmin protein immunoreactivity at the vascular level in the hindbrain of a *Cp*+/+ infected mouse; *Cp*-/- mice was used as control for the primary antibody. (C) Dark-field photomicrographs showing representative hybridization signals of Nfkbia or Selp riboprobes on hindbrain sections of *Cp*+/+ or -/- mice killed at the early phase of infection (36–48 h post-infection). (D) High magnification photomicrographs depicting the expression pattern of Selp mRNA associated with large blood vessels and not in cells lining the meninges or the ventricle. (E) Semi-quantitative analysis of Nfkbia and Selp mRNA levels O.D. Student's t-test was used to compare *Cp*+/+ and *Cp*-/- means (see text for details); * p<0.05. Abbreviations: bv, blood vessel; Cb, cerebellum; IC, inferior colliculus; m, meninges.

Since blood vessels are strategically positioned to produce Cp protein during systemic infection, we evaluated whether this APR modulates gene expression in vascular-associated elements during meningitis. *S. pneumoniae* infection was used to test a possible role of Cp in this context. No difference was found in the survival curves of the three genotypes following *S. pneumoniae* inoculation (Fig. 7A). Nevertheless, Cp was strongly induced in brain blood vessels of infected *Cp*+/+ mice (Fig. 7B). We then determined Nfkbia (IκBα) and Selp (P-Selectin) gene expression; Nfkbia expression is a general index of NF-κB activation, which seems quite similar in the cerebrovascular system of the three genotypes (Fig. 7C, F). In contrast, Selp transcript was mainly induced in large blood vessels (Fig. 7 D, E, F) and its expression level was higher in the brain of *Cp*-/-mice. The time point 48 h was used for this comparison, because the variability among mice was high from day 3 and the number of animals that exhibited clinical signs was irregular between the genotypes and this variability complicated the interpretation of the results. Forty-eight h after inoculation, the number of animals with clinical signs was equivalent in both *Cp*+/+ (n = 6) and *Cp*-/-(n = 7) group, but not in the group of *Cp*+/-mice (n = 3). Since the highest variance was associated with *Cp*+/-genotype, we removed this group from the statistical analysis to avoid misrepresentation and substantial reduction of power. In this regard, Selp mRNA levels were significantly different between *Cp*+/+ and *Cp*-/-according to student's t-test (p<0.05), but not when ANOVA is used to compare the three genotypes. Nevertheless, a significant linear trend between Selp mRNA levels and *Cp* deficiency was reached (p<0.05) when assigning 0, 1 and 2 values to *Cp*-/-, +/-and+/+genotypes, respectively. These statistical tests did not reveal any significant changes in Nfkbia mRNA levels among two or three genotypes.

## Discussion

### New roles for GR signaling during acute neuroinflammation

The capacity of GR to repress NF-κB/AP-1 activity and gene transcription is believed to be a key intracellular process mediating the powerful anti-inflammatory properties of synthetic and endogenous GCs. Such a mechanism was elegantly explored by gene profiling using cell-culture system and the GR agonist Dexamethasone (Dex) [12]. Surprisingly we found that blocking endogenous GR signaling exacerbated the expression of a small and restricted set of inflammatory genes induced by LPS in the CNS. Curiously, most of these upregulated genes were found in the contralateral side, probably reflecting saturation in the ipsilateral side and spreading to distal sites. Genes with this behavior belong to the chemokine network and then to an increased recruitment of inflammatory cells. Interestingly, the effects of RU486 and LPS was found for genes linked to adaptive immunity Cd83 and Tnfsf9 [13], [14]. Ablation of glucocorticoid signaling also intensified induction of transcription regulator Litaf and Zfp36 and the regulator of JAK-STAT pathway Pim-1 and Socs3.

Therefore our results do not rule out the possibility that GR antagonism increases the inflammatory reaction in the CNS, especially at later time-points. These data do suggest that genetic profile is no longer respected in the absence of GR signaling on the cerebral immune system. Although the effects of GRs on the regulation of genes encoding APRs were expected [15], this was not the case for those implicated in host defense. In this regard, a recent study has demonstrated that combination of Dex treatment can lead to an unpredicted genetic response to LPS [16] or enhance expression of immune genes in peripheral blood monuclear cells [17]. Hence, it is very likely that the role of GCs on expression of inflammatory genes depends on various factors, including cell/tissue type, pharmacological strategy and use of *in vivo* or *in vitro* models. Some of our results may be in sharp contrast to those previously reported because the paradigm used here to access the role of endogenous glucocorticoids does not block the inflammatory process.

Our gene profiling data indicated that endogenous GCs are required to coordinate the ability of LPS for inducing expression of genes involved in the viral and interferon response. These include components of viral dsRNA sensing pathway Ifih1 (MDA-5), Ikbke (iKK/IKKε) and Irf7 [18]. In addition, we have identified a set of genes that participate in the antiviral network of interferon response initiated by Stat1/2, namely Isgf3g (IRF-9), Oas1a and Eif2ak2 (PKR) [19]. Expression of genes encoding GTPases was also dependent on GR signaling. Although the functions of these molecules remain largely unknown, a role for Irgm (Ifi1/LRG-47) was recently described in the generation of large autolysosomal organelles that eliminate intracellular *Mycobacterium tuberculosis* [20]. Therefore, our results may help explaining why Dex treatment is beneficial in tuberculosis meningitis, which is not associated with attenuation of the immune response [21]. In addition, a GC replacement therapy has been shown to have beneficial effects in a context of septic shock [22]. Importantly, impaired gene expression by GR interference may increase infection susceptibility, which was recently found in few cases of RU486-treated women [23].

Although GR signaling is necessary to support innate immune response gene expression, our results should not be interpreted as a newly described pro-inflammatory role of GCs. Many recent studies pointed that GC exposure may increase CNS inflammation and Sorrells and Sapolsky suggested a conciliatory manner to interpret both anti- and pro-inflammatory effects of GCs based on early, delayed, acute or chronic regimen [24]. We suggest careful attention in order to dissociate gene expression from inflammation. Several genes that depend on GR to be induced by LPS play important roles in terms of cell differentiation, acute phase response, extracellular matrix organization and interferon response, all of which potentially helping to resolve inflammation rapidly. The lack of this gene subset in association with the exacerbated expression of genes identified here as GR repressed (chemokines, Tnfsf9, Cd83, etc.) can clearly result in increased inflammation at latter time-points and in the emergence of a detrimental microglial/macrophage phenotype.

It is interesting to note that many regulated genes that exhibited a particular profile (i.e., RU486 prevented their downregulation to LPS) are related to brain development and not necessarily to myeloid functions. These data suggest alternative mechanisms underlining the actions of GC and TLR4 signaling on gene repression in the CNS. Our factorial design also allowed us to report a list of genes that follows a GR-blockage main effect, revealing genes that respond to RU486 and are not changed by the inflammatory reaction.

### Anatomical findings regarding GR-modulated inflammatory gene expression

As depicted by the figure 3 and 4, a very distinct anatomical distribution was found for most regulated genes by the treatment combining RU486 to LPS. Such finding, which is further illustrated in co-localization experiments (Figure S3), implies that these transcripts may play very different roles despite having similar regulatory processes. While it is possible to infer an immune role for Tnfsf9 and Cd83 in myeloid cells, the role of Gem in cells lining the meninges must be further explored. Serpina3n (a mouse homologue of α1-antichimotrypsin), Cd44, and Ch25h expression levels were also verified, because of the novelty of the gene array results and their possible involvement in the physiopathology of Alzheimer's disease [25]–[27]. Unbalance of the inflammatory and GC response is also a feature of the disease [6], [28]. Stat1 induction in neurons was startling, because it is usually a gene related to viral and adaptive immune responses [29] and here we found a selective neuronal transcriptional activation during innate immunity. The lack of Il1b induction following RU486/LPS administration was also quite intriguing, although increased expression of this cytokine following GC administration has been reported [30]. Careful interpretation must be taken regarding this particular transcript, because *in situ* hybridization could not clearly corroborate this result.

The endotoxin was able to downregulate a number of genes in a GR-dependent manner. These include C1qa and Hexb genes, but the meaning of their localized suppression during neuroinflammation has yet to be determined. Inhibition of the classical complement pathway could be a natural mechanism to avoid a precipitated antibody mediated cell lysis, whereas Hexb may have uncharacterized immune properties modulating neuronal functions. Indeed, Hexb gene disruption in mice leads to neurological deficits, which can be partially prevented by the transplantation of bone marrow stem cells taken from wild type mice [31].

### Cp links glucocorticoid and inflammation to iron homeostasis and brain protection

The gene profiling results clearly pointed out that APR gene induction was a prominent feature of the ability of GRs to modulate the inflammatory transcriptional programs. The role of APRs during infection is not well understood, but a subset of genes is implicated in iron homeostasis. For instance, Lcn2 plays an important role in iron acquisition during infection [32]. Because iron unbalance is implicated in demyelinating and neurodegenerative diseases [33], [34] and aceruloplasminemia leads to brain, liver and pancreas iron accumulation with concomitant neuronal damages [35], [36], Cp is a putative candidate to mediate neuroprotection during inflammation. Surprisingly, murine models of aceruloplasminemia accumulate iron, but they do not develop the neurological features of the disease [37], [38]. Iron accumulation in human disease and murine models led to the inference that Cp is important to release iron from the cellular pool, especially mediated by a membrane glycosylphosphatidylinositol-form of this ferroxidase [39]. Conversely, it has been suggested that extracellular Cp mediates cellular iron uptake and iron absorption during stress [40]. Our findings suggest that an acute inflammatory reaction leads to tissue iron accumulation that persists through the course of the immune challenge. Cp is not required for LPS mediated iron (III) deposition, but this protein seems to play a role avoiding precipitated iron unbalance.

The physiological relevance of this process has yet to be determined, but it could be related to limit the iron uptake by bacteria or to avoid an exacerbated accumulation of reactive iron (II) levels. The data that LPS caused CNS injury in *Cp*-/-mice and not their control littermates are quite interesting, because they underline the critical neuroprotective role of Cp in an inflammatory context. We propose that Cp produced by microglial cells is released to the extracellular space for controlling iron homeostasis in this critical period, when white matter elements are more vulnerable. Hence, these findings support an important new role for Cp in mediating neuroprotection in the course of an acute innate immune process. Such neuroprotective properties of Cp are not associated with changes in microglial reactivity to LPS.

We also observed that Cp is highly expressed in cells lining the blood vessels. In consequence, it was attractive to test the hypothesis that Cp could be a mediator of barrier function, protecting the brain against circulating pathogens or potentially damaging molecules derived from their infection. *S. Pneumoniae* is a leading cause of meningitis worldwide. It is proposed that high levels of bacteria in the blood targets the endothelium in order to reach brain compartments [41]. We then used a model of peripheral inoculation passive of meningitis. Although no changes in terms of susceptibility were observed in the course of infection among genotypes, *Cp*-/-exhibited a very different vascular reactivity to the circulating bacteria. In fact, Cp deficiency was associated with a robust expression of Selp transcript in large blood vessels and arteries. Selp controls key events related to leukocyte cell recruitment and this adhesion molecule is a determinant modulator of vascular permeability to bacterial elements [42]. This highlights another new function of Cp in stabilizing neurovascular responses in the course of pneumococcal infection.

### Concluding Remarks

Endogenous GCs play a critical role for modulating the innate immune response in the CNS. Ablation of GR signaling exacerbated the expression of a small subset of genes, which are implicated in cell recruitment and prolonged immune activity. Surprisingly, expression of many genes related to host defense were critically impaired upon RU486 treatment, a mechanism that could explain unusual bacterial infections in individuals taking this compound as abortion pill and the beneficial effects of an acute therapy with GCs. This gene profiling experiment led us to investigate the role of Cp in the course of an acute LPS challenge. Experiments using *Cp*-deficient mice generated exciting data regarding the ability of acute-phase proteins to make the difference between cell survival and death during a natural and timely sensitive innate immune reaction in the CNS.

## Materials and Methods

### Animal experimentation

Adult male C57Bl/6 (body weight, 25–29 g; Charles River Canada, St. Constant, Québec, Canada) were acclimated to standard laboratory condition. Ceruloplasmin-deficient mice (*Cp*-/-) in C57Bl/6 background [38] were backcrossed two rounds with female C57Bl/6 mice. The backcrossed heterozygote mice (*Cp*+/-) were used to generate *Cp*+/+, +/- and -/-mice. These mice were genotyped by PCR of DNA isolated from ear punching. Animal breeding and experiments were conducted according to Canadian Council on Animal Care guidelines, as administered by the Laval University Animal Care Committee. Efforts were made for minimizing animal number utilization; 3 to 6 animals per group were used in each experimental group, except for infection protocol (19 to 20 mice per group).

### Inflammatory treatment

In protocols for a comparative study of the effects of endogenous GCs, C57Bl/6 mice received an i.p. injection of vehicle (DMSO-50 µl) or RU486 (50 mg/kg b.w.) and were submitted to surgery 4 h later. Just after the surgery the animals received a second i.p. injection of vehicle or RU486 to guarantee high brain levels of the drug. In a second protocol mice received different doses of RU486 (5, 50 and 200 mg/kg b.w.) in similar conditions in order to test dose-dependent effects. The mice receiving intraparenchymal injections were anesthetized with a mixture of ketamine and xylazine and placed in a stereotaxic apparatus (David Kopf Instruments). The right caudate putamen was reached, using a small cannula (28 gauge; Plastic One) at the coordinates 0.0 mm anteroposterior, −2.0 mm lateral, and −3.0 mm dorsoventral according to a mouse brain atlas [43]. The coordinates were selected on the basis of previous experiments showing a robust hybridization signal and reliable pattern of cytokine gene expression over the ipsilateral cerebral cortex, hippocampus, corpus callosum and basal ganglia following a single bolus of immune ligand. The animals received an infusion of either sterile pyrogen-free saline (1 µl) or LPS (2.5 µg; from Eschericia coli; serotype O55:B5; Sigma L2880) over 2 min by means of a microinjection pump. Animals were killed 12 h after the injection for the microarray gene profiling and validation experiments. Another group of *Cp*-deficient mice and respective controls (+/-, -/-) were killed 2 and 5 days after intrastriatal (IS) infusion with saline or LPS.

For gene array and qPCR assay, mice were anesthetized under isofluorane and blood was drawn via cardiac puncture before head decapitation. Brains were removed rapidly from the skulls and placed in cold phosphate buffered saline (PBS) solution. A brain region defined by dashed lines in figure 1 (limited at plane anteroposterior +1.5 to −1.5 and dorsoventral −4.0) was dissected, separated in ipsilateral and contralateral sides, and quickly immersed in liquid nitrogen. The tissue was stored at −80°C until RNA extraction was performed. The dissected area was estimated to contain the corresponding potential injured area and was restricted to avoid dilution of the sample with RNA from non-relevant brain regions.

For *in situ* hybridization histochemistry, histology and immunolabeling, animals were deeply anesthetized via an intraperitoneal injection of a mixture of ketamine hydrochloride and xylazine and then rapidly perfused transcardially with 0.9% saline, followed by 4% paraformaldehyde in PBS, pH 7.6, at 4°C. Brains were removed rapidly from the skulls, postfixed for 2–4 d, and then placed in a solution containing 20% sucrose overnight at 4°C. The frozen brains were mounted on a microtome (Reichert-Jung) and cut into 25-μm coronal sections. The slices were collected in cold cryoprotectant solution and stored at −20°C.

### Streptococcus Pneumoniae infection

A virulent *S. Pneumoniae* strain capsular type 6A DBL6A was used in an experimental model of pneumococcal infection passive of meningitis. The mice (n = 19 for *Cp*+/- and -/-; n = 20 for *Cp*+/+) were anesthetized and received an i.p. injection of the working suspension (50–100 C.F.U./ml) in a 100 µl volume [see supplementary materials and methods (Text S1) for bacteria suspension preparation]. Animals were assigned to receive the injections in a random manner. Whenever mice presented clinical and/or dehydration signs, Ringer's lactate and/or buprenorphine were administrated to relief discomfort. Once animals showed clearly signs of irreversible illness or neurological impairment, they were sacrificed by cardiac perfusion as described above except that PFA was prepared in 0.1 M borax buffer, pH 9.5.

### RNA isolation and Oligonucleotide array

The RNA was isolated using Trizol reagent (Invitrogen) following manufacture's protocol and 10 µg of total RNA was used for cDNA synthesis. Converted cDNA using a T7-oligo-d(T)24 primer were used to generate biotinylated cRNA, the latter was fragmented before hybridization, as described in [44]. The quality of total RNA, cDNA synthesis, cRNA and cRNA fragmentation were all monitored by micro-capillary electrophoresis (Bioanalizer 2100, Agilent Technologies). cRNA probes were hybridized onto MOE403A mouse Genechip (Affymetrix) and processed using an Affymetrix GeneChip Fluidic Station 400. Staining was performed with streptavidin-conjugated phycoerythrin (SAPE) followed by amplification with a biotinylated anti-streptavidin antibody and by a second round of SAPE solution. The GeneChips were scanned using an Agilent GeneArray Scanner (Agilant Technologies).

### Oligonucleotide array statistical analysis

After careful quality control to discard abnormal samples according to multiple evidences (4 chips from different groups were removed), a total of 37 chips were used for oligonucleotide array analysis [one chip per biological sample; 8 groups (contralateral dmso/saline, dmso/LPS, RU486/saline, RU486/LPS and ipsilateral dmso/saline, dmso/LPS, RU486/saline, RU486/LPS) with 4–6 biological replicates each]. Expression values from the CEL files generated from scanning were obtained using RMA algorithm [45], available at http://www.bioconductor.org. The expression values were also inspected with GeneSpring software (Silicon Genetics). Statistical analysis was performed considering a factorial linear model according to the methods implemented in Limma package [46] (R project packages are available at http://www.cran.r-project.org). For details on differential gene expression and Gene Ontology (GO) analysis, please refer to text S1. The data discussed in this publication have been deposited in NCBIs Gene Expression Omnibus (GEO, http://www.ncbi.nlm.nih.gov/geo/) and are accessible through GEO Series accession number GSE6509.

### Quantitative Polymerase Chain Reaction (qPCR)

Real-time RT-PCR was performed as described in [47], for further details refer to text S1.

### cRNA probes and in situ hybridization

Plasmids were linearized and the sense and antisense ^35^S-labeled riboprobes were synthesized as described before ([48], refer to text S1 for details). Hybridization histochemical localization of the different transcripts was performed on every 6th or 12th section of the entire rostrocaudal extent of each brain. The sections were exposed at 4°C to x-ray films (Biomax; Kodak). The slides were thereafter defatted in xylene, dipped into NTB-2 nuclear emulsion (Kodak).

### Combination of immunohistochemistry with in situ hybridization

Immunohistochemistry was combined with the *in situ* hybridization histochemistry protocol to determine the types of cells that express Cd44, Cd83, Cp, Lcn2, Saa3, Stat1 and Tnfsf9 transcripts. Anti-CD31 was used to stain the endothelial cells of the vasculature, whereas anti-ionized calcium binding adapter molecule 1 (IBA1), anti-glial fibrillary acidic protein (GFAP) and anti-neuron specific nuclear protein (NEUN) labeled cells of myeloid (macrophages and microglia), astroglial and neuronal lineages, respectively. Dual labeling was performed as described in [48]; rat anti-mouse CD31 (BD Biosciences Pharmingen), rabbit anti-human IBA1 (Wako), monoclonal anti-mouse GFAP (Chemicon) and monoclonal anti-mouse NEUN (Chemicon) were diluted at 1∶500, 1∶1500, 1∶1000 or 1∶2000, respectively.

### Iron, Myelin and, FluoroJade B (FJB) staining

Iron (III) staining was performed on free-floating sections as a described improved method for Perls' stain [49]. Specificity of dark brown deposits was verified in replacing potassium ferrocyanide for potassium ferricyanide [for iron (II) detection], which failed to reveal such staining. Myelin content and byproducts were determined via Sudan Black B (SBB) staining, whereas neuronal cell death was investigated by means of the Fluoro-Jade B (FJB) method, as previously described [9], [11].

### Immunohistochemistry and Immunofluorescence

Immunolabeling was performed on free-floating sections following standard procedures detailed in text S1. Cp immunohistochemistry staining specificity was verified using brain sections of *Cp* deficient mice. Double immunofluorescence staining was performed in sequential manner; primary antibody against Cp detection with Alexa488-conjugated goat anti-rabbit was followed by incubation of primary antibodies against CD31, GFAP, IBA1, or MAC-2 that were detected with immunoglobulin antibodies conjugated to Cy3.

Photomicrographs were taken with the same exposure time using a digital camera (QIMAGING) mounted directly on a microscope (Nikon Eclipse 80i). Confocal laser scanning microscopy was performed with a BX-61 microscope equipped with the Fluoview SV500 imaging software 4.3 (Olympus America Inc). Confocal images were acquired by sequential scanning using a two-frame Kalman filter and a z-separation of 1 µm.

### In situ Hybridization signal Analysis

Semi-quantitative analyses of hybridization signals were performed as described previously [11], but differential (ipsilateral–contralateral) optical density (O.D.) was not used for transcripts that had signals extending largely to the contralateral side. The average O.D. after background correction was used for these particularly widely spread mRNAs. Genes with restricted, constitutive or unusual pattern of expression were quantified accordingly to their characteristics to avoid miscalculation or underestimation of signals. Samples from *S. pneumoniae* infection protocol were analyzed at the level of hindbrain. Quantification of area without Plp1 transcript signal (demyelinated regions and areas associated with lesions) was performed as previously described [11].

### Estimation of Area and Volume by Stereology

Stereological analysis was performed as previously described [11]. Systematically sampled sections were stained for iron III or Fluorojade B staining. The area associated with dark Iron III deposits was traced and the total area values for each sample were acquired using Neuroexplorer software (MicroBrightField). Volume of brain regions showing FJB-positive staining was estimated by the Cavalieri method (50-µm point grid) using Neurolucida Stereo Investigator software (Microbrightfield).

### Image processing

Photomicrographs were processed to enhance contrast and image quality using Adobe Photoshop 8 (Adobe Systems) and were assembled using Adobe Illustrator (Adobe Systems). The image edition was kept to a minimum to avoid artifacts. Pictures representing different groups received equivalent image treatment.

### Statistical Analysis

Comparison of group means was performed using an one-way ANOVA followed by a Tukey's HSD procedure as post hoc multiple comparisons test. Two or three factor ANOVA was performed using Bonferroni's correction when applicable and also according to presence or absence of interaction between factors. Student's t-test was applied for comparison between two groups according to homogeneity of variances. All the analyses were carried out with SPSS software version 11.0, except for Logrank test and Logrank test of trend to compare survival curves, which were performed using Prism 4 software.

## Supporting Information

Dataset S1 Excel Spreadsheet; LPS modulated genes
(0.06 MB XLS)

Dataset S2 Excel Spreadsheet; LPS Vs LPS/RU486 differential gene expression
(0.06 MB XLS)

Figure S1 Gene expression exploration data
(2.86 MB PDF)

Figure S2 C1qa and Serping1 expression profile
(0.19 MB PDF)

Figure S3 Cellular site of transcript synthesis
(0.42 MB PDF)

Figure S4 Cp expression and IBA1-Vs MAC2-positive cells
(0.44 MB PDF)

Figure S5 Time-dependent extracellular Cp expression
(0.31 MB PDF)

Text S1 Supplementary Materials and Methods
(0.18 MB PDF)
