# Supplementary material for: Genes Involved in the Balance between Neuronal Survival and Death during Inflammation
Source: PLoS One. 2007 Mar 21;2(3):e310. doi: 10.1371/journal.pone.0000310 (PMC1819560; doi:10.1371/journal.pone.0000310)
Supplement: Figure S1 — Gene expression exploration data (2.86 MB PDF) [file pone.0000310.s003.pdf]

Figure S1A -Genes Upregulated by LPS

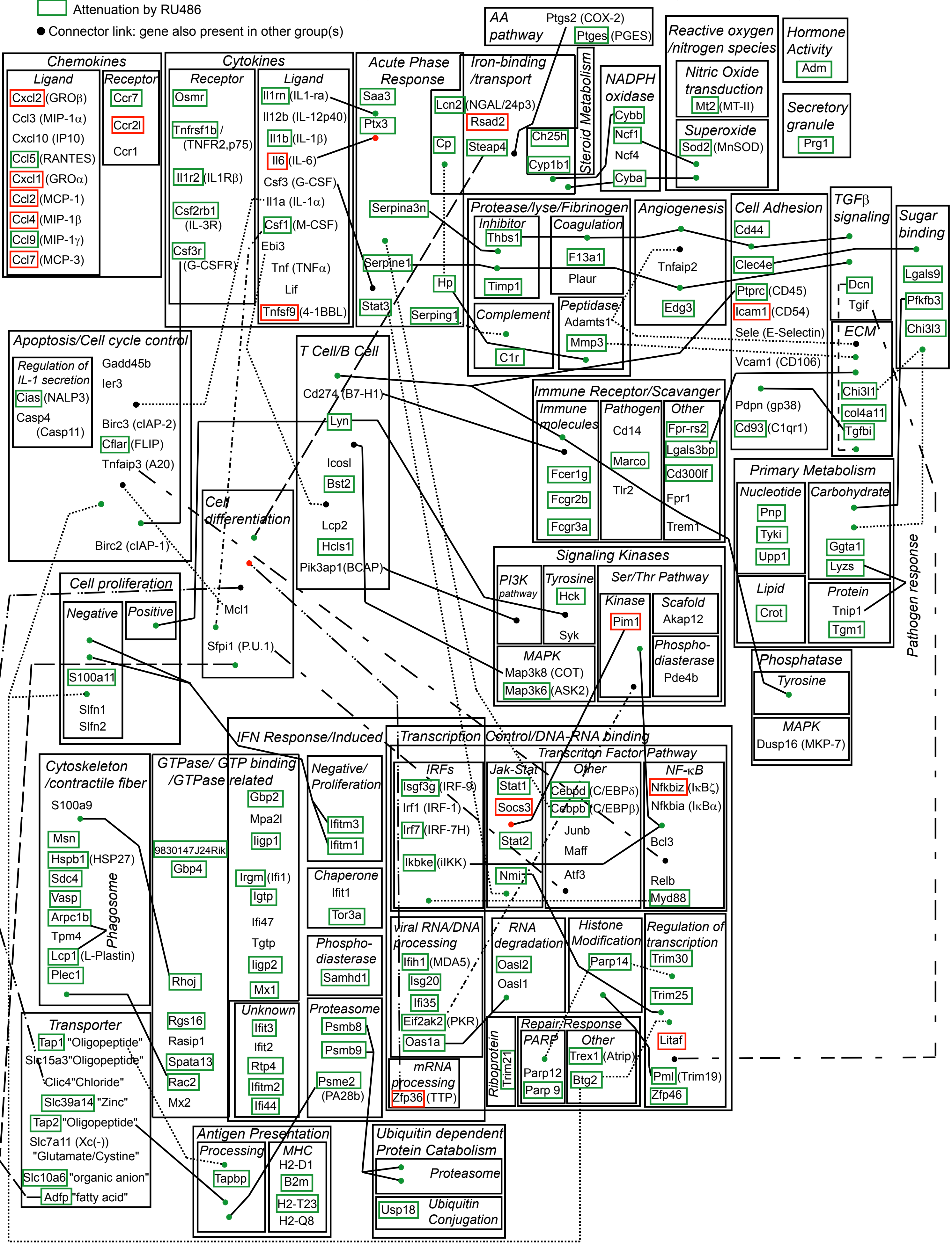

Figure S1B -Genes Downregulated by LPS

LPS Downregulation attenuated by RU486

● Connector link: gene also present in other group(s)

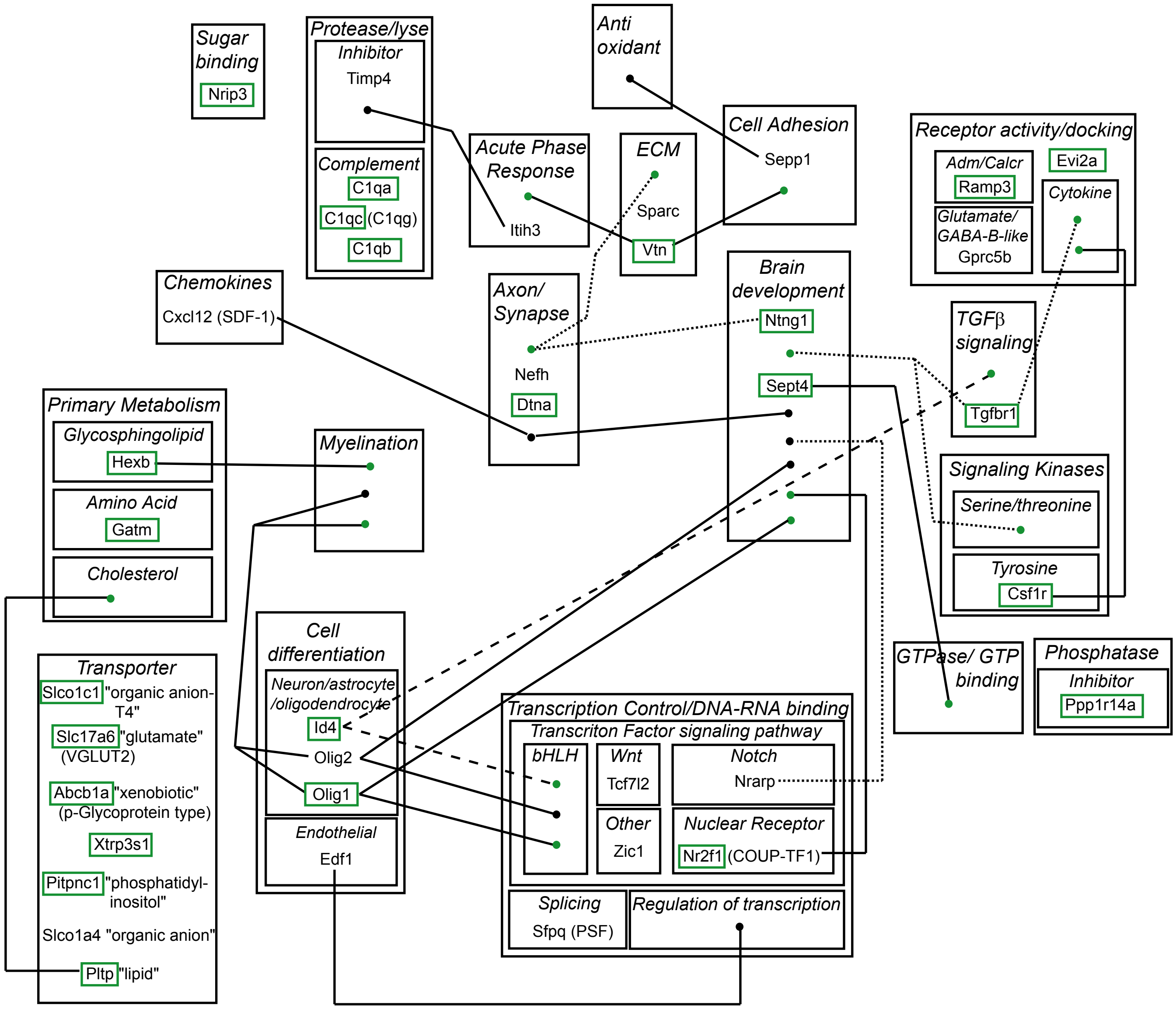

Genes Upregulated by LPS only in presence of RU486

Marker of mature dendritic cell — Cd83

Adaptive Immunity

small GTPase signal transduction — Gem

Calcium channel modulation

cytoskeleton

Transcription factor CNC-bZip family — Bach1

Repress Maf recognition element (MARE) when dimerized to Mafk

Heme signaling

Figure S1C - RU486 main effect probe sets

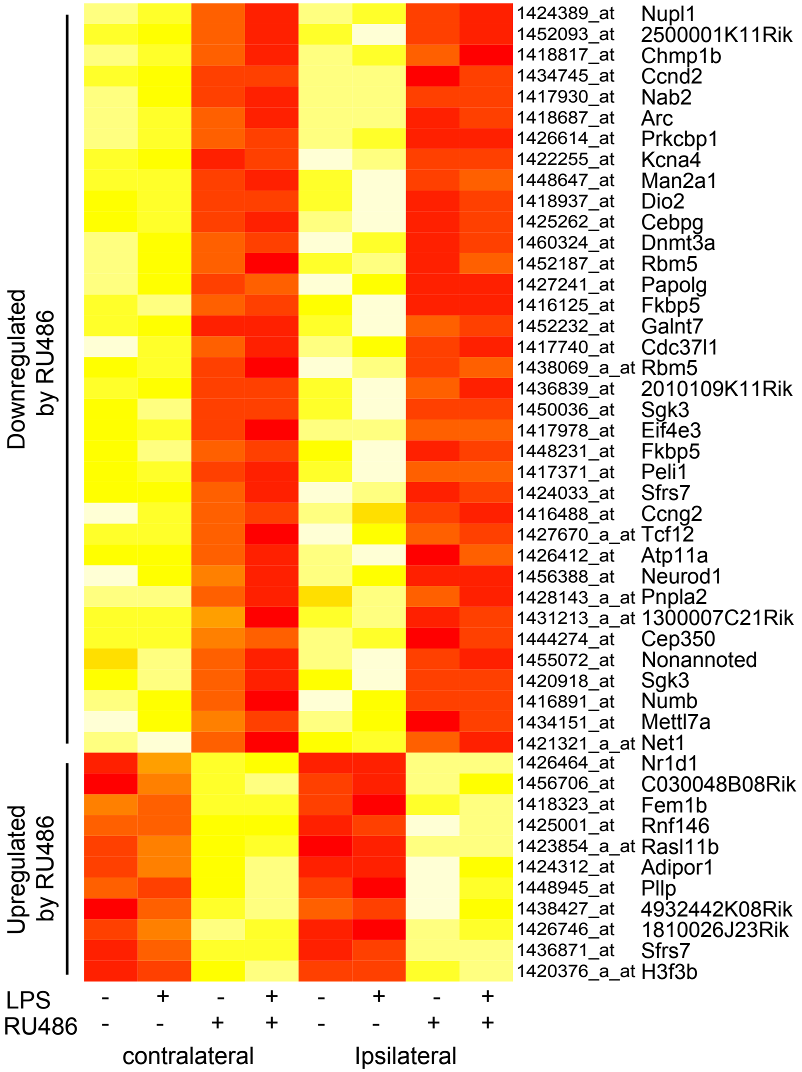

Figure S1D - RU486 Main effect 2-contrast plot

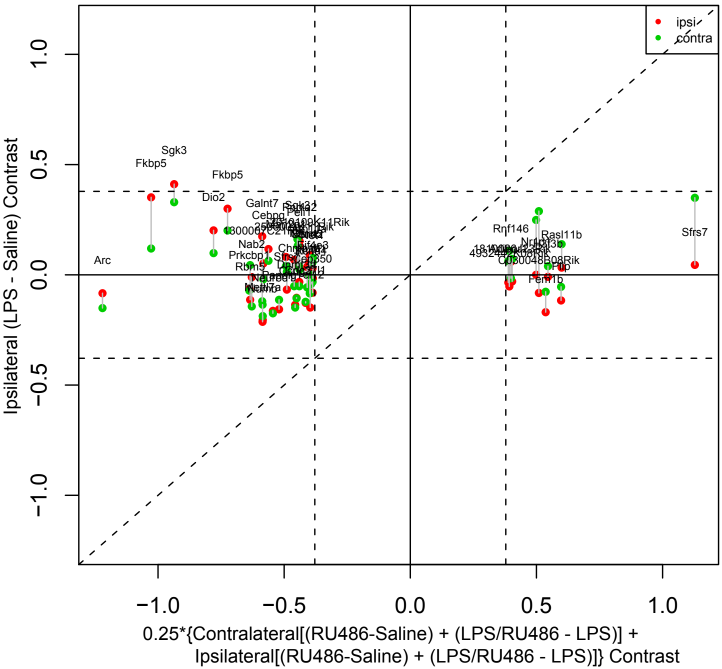

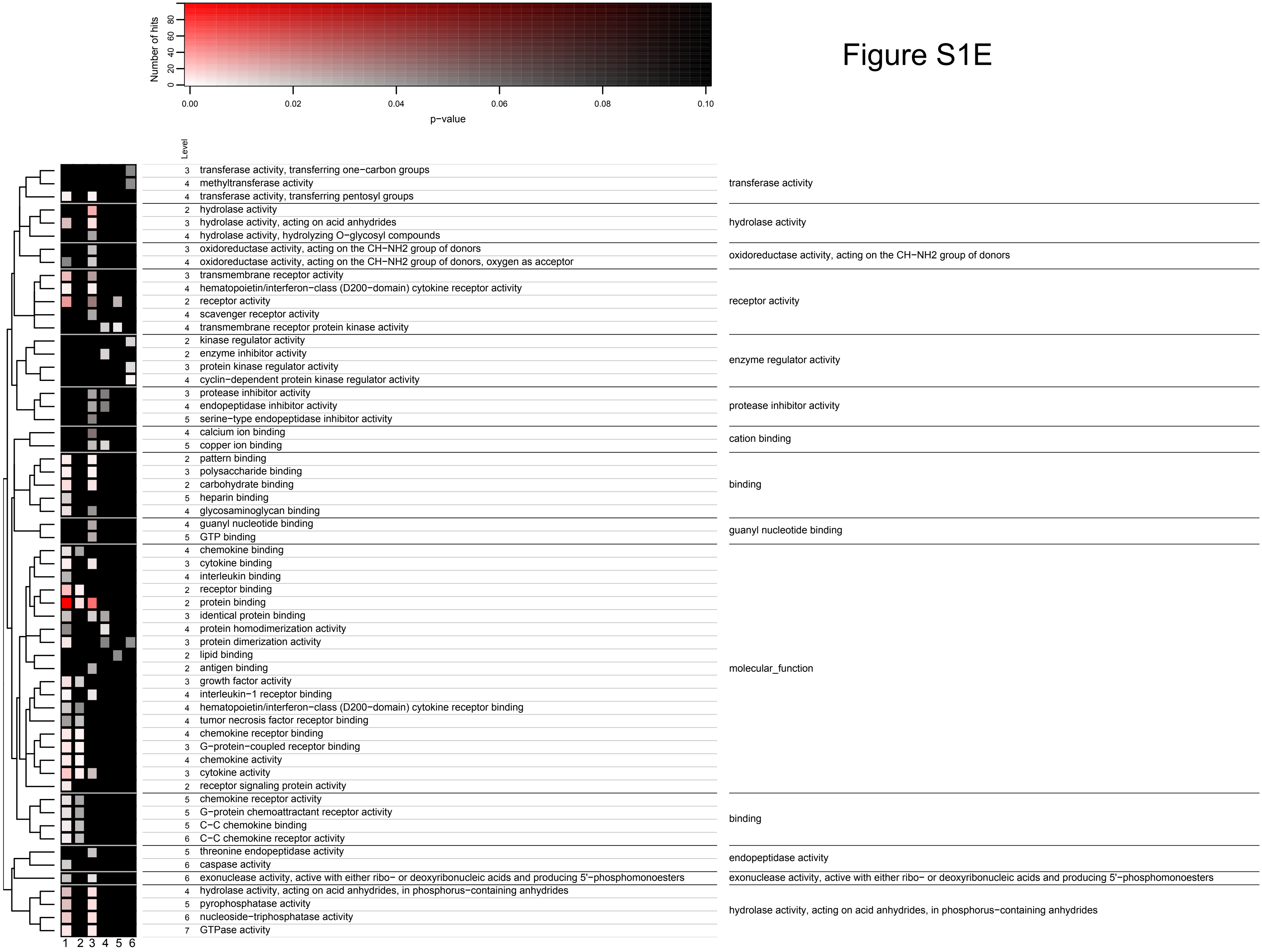
Number of hits
0
20
40
60
80
0.00
0.02
0.04
0.06
0.08
0.10
p−value
Level
3 transferase activity, transferring one−carbon groups
4 methyltransferase activity
4 transferase activity, transferring pentosyl groups
2 hydrolase activity
3 hydrolase activity, acting on acid anhydrides
4 hydrolase activity, hydrolyzing O−glycosyl compounds
3 oxidoreductase activity, acting on the CH−NH2 group of donors
4 oxidoreductase activity, acting on the CH−NH2 group of donors, oxygen as acceptor
3 transmembrane receptor activity
4 hematopoietin/interferon−class (D200−domain) cytokine receptor activity
2 receptor activity
4 scavenger receptor activity
4 transmembrane receptor protein kinase activity
2 kinase regulator activity
2 enzyme inhibitor activity
3 protein kinase regulator activity
4 cyclin−dependent protein kinase regulator activity
3 protease inhibitor activity
4 endopeptidase inhibitor activity
5 serine−type endopeptidase inhibitor activity
4 calcium ion binding
5 copper ion binding
2 pattern binding
3 polysaccharide binding
2 carbohydrate binding
5 heparin binding
4 glycosaminoglycan binding
4 guanyl nucleotide binding
5 GTP binding
4 chemokine binding
3 cytokine binding
4 interleukin binding
2 receptor binding
2 protein binding
3 identical protein binding
4 protein homodimerization activity
3 protein dimerization activity
2 lipid binding
2 antigen binding
3 growth factor activity
4 interleukin−1 receptor binding
4 hematopoietin/interferon−class (D200−domain) cytokine receptor binding
4 tumor necrosis factor receptor binding
4 chemokine receptor binding
3 G−protein−coupled receptor binding
4 chemokine activity
3 cytokine activity
2 receptor signaling protein activity
5 chemokine receptor activity
5 G−protein chemoattractant receptor activity
5 C−C chemokine binding
6 C−C chemokine receptor activity
5 threonine endopeptidase activity
6 caspase activity
6 exonuclease activity, active with either ribo− or deoxyribonucleic acids and producing 5'−phosphomonoesters
4 hydrolase activity, acting on acid anhydrides, in phosphorus−containing anhydrides
5 pyrophosphatase activity
6 nucleoside−triphosphatase activity
7 GTPase activity
1 2 3 4 5 6
transferase activity
hydrolase activity
oxidoreductase activity, acting on the CH−NH2 group of donors
receptor activity
enzyme regulator activity
protease inhibitor activity
cation binding
binding
guanyl nucleotide binding
molecular_function
binding
endopeptidase activity
exonuclease activity, active with either ribo− or deoxyribonucleic acids and producing 5'−phosphomonoesters
hydrolase activity, acting on acid anhydrides, in phosphorus−containing anhydrides

Figure S1E

Figure S1F

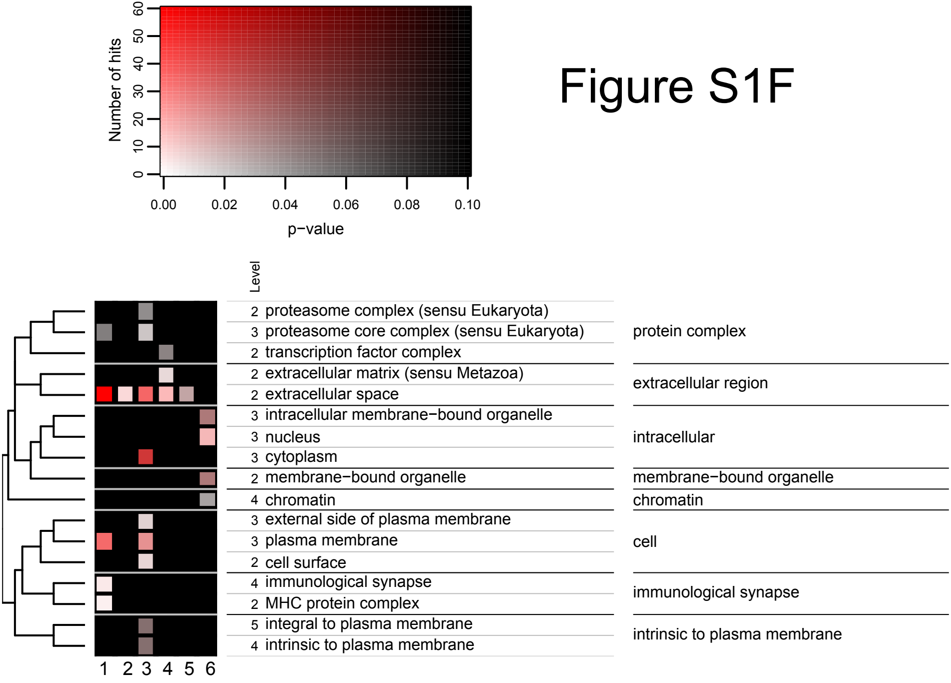

Number of hits
0
10
20
30
40
50
60
0.00
0.02
0.04
0.06
0.08
0.10
p−value
Level
2 proteasome complex (sensu Eukaryota)
3 proteasome core complex (sensu Eukaryota)
2 transcription factor complex
2 extracellular matrix (sensu Metazoa)
2 extracellular space
3 intracellular membrane−bound organelle
3 nucleus
3 cytoplasm
2 membrane−bound organelle
4 chromatin
3 external side of plasma membrane
3 plasma membrane
2 cell surface
4 immunological synapse
2 MHC protein complex
5 integral to plasma membrane
4 intrinsic to plasma membrane
protein complex
extracellular region
intracellular
membrane−bound organelle
chromatin
cell
immunological synapse
intrinsic to plasma membrane
1 2 3 4 5 6

Figure S1A – Diagram representing relevant functional categories of genes upregulated by LPS. Genes selected by oligonucleotide array statistical analysis were grouped in each category box and vertically organized according to a fold change sorting (in each box). Such classification was organized according to authors' interpretation of the results. For non-biased statistical inference of gene function readers should refer to Figure 2 of the main manuscript. Gene symbols surrounded by green boxes indicate activated genes that had a significant lower expression when animals received RU486 pre-treatment before LPS challenge. Red boxes indicate genes that presented exacerbated expression by RU486 in combination to LPS.

Figure S1B – Diagram representing relevant functional categories of genes downregulated by LPS or exclusively induced in response to LPS/RU486 treatment. Genes selected by oligonucleotide array statistical analysis were grouped in each category box and vertically organized according to a fold change sorting (in each box). Such classification was organized according to authors' interpretation of the results. Please see the Figure 2 (main manuscript) for non-biased statistical inference of gene function. Genes highlighted by green boxes are those locally repressed by LPS treatment, but their repressive effect is significantly attenuated by RU486 pre-treatment.

Figure S1C – List of RU486 main effect probe sets. The list is organized following a heat map. Lower expression values coded in red and higher values coded in white/yellow.

Figure S1D – A 2-contrast plot of genes selected as differently expressed according to RU486 main effect. The abscissa axis represents the difference (contrast) between all samples treated with RU486 treatment and those treated with DMSO (vehicle) in RMA expression levels;

ordinate axis represents the LPS *vs*. Saline contrast. Red dots represent ipsilateral contrast value; green dots, represent contralateral contrast values.

Figure S1E – Plot showing significant molecular function (hypergeometric distribution) associated with six different lists of differently expressed genes (1 - LPS upregulated, 2 - LPS upregulation exacerbated by RU486, 3 - LPS upregulation prevented by RU486, 4 - LPS downregulated, 5 - LPS dowregulation prevented by RU486 and 6 - RU486 main effect). Hierarchical clustering of the Gene Ontology nodes was performed as described in experimental procedures supplementary file. A color/intensity code assigns number of genes and p-value for each molecular function associated with the lists' heat map.

Figure S1F –The plot depicts significant cellular components (hypergeometric distribution) associated with six different lists of differently expressed genes (1 - LPS upregulated, 2 - LPS upregulation exacerbated by RU486, 3 - LPS upregulation prevented by RU486, 4 - LPS downregulated, 5 - LPS dowregulation prevented by RU486 and 6 - RU486 main effect). Hierarchical clustering of the Gene Ontology nodes was performed as described in experimental procedures supplementary file. A color/intensity code assigns number of genes and p-value for each cellular component associated with the lists' heat map.
