# Supplementary material for: Genes Involved in the Balance between Neuronal Survival and Death during Inflammation
Source: PLoS One. 2007 Mar 21;2(3):e310. doi: 10.1371/journal.pone.0000310 (PMC1819560; doi:10.1371/journal.pone.0000310)
Supplement: Figure S2 — C1qa and Serping1 expression profile (0.19 MB PDF) [file pone.0000310.s004.pdf]

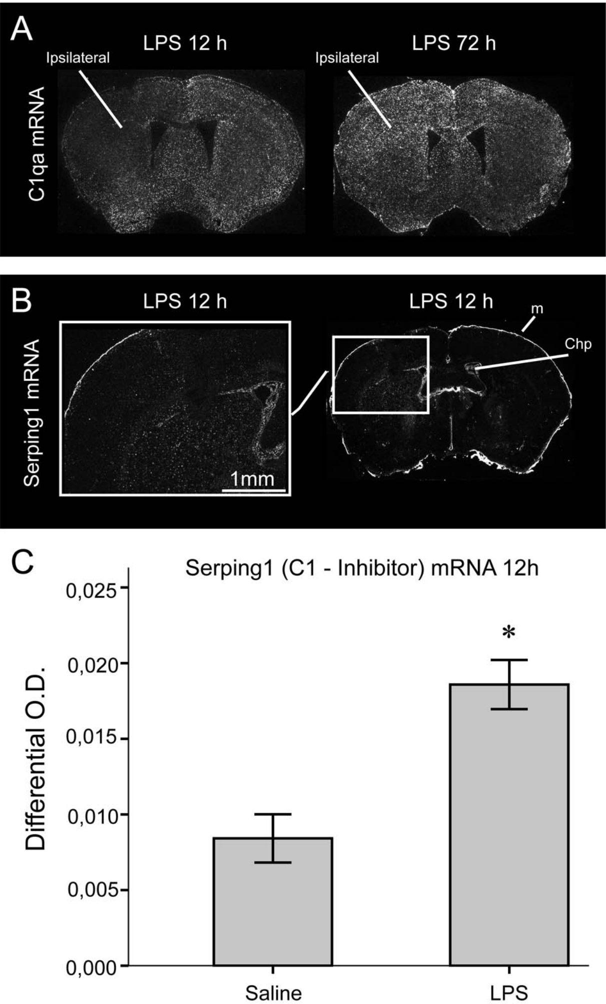

A
LPS 12 h
LPS 72 h
Ipsilateral
Ipsilateral
C1qa mRNA
B
LPS 12 h
LPS 12 h
m
Chp
Serping1 mRNA
1mm
C
Serping1 (C1 - Inhibitor) mRNA 12h
Differential O.D.
0,025
0,020
0,015
0,010
0,005
0,000
*
Saline
LPS

Figure S2 – Dynamic of classical complement pathway in the course of an acute LPS challenge. (A) C1qa mRNA expression following 12 or 72 h post LPS (2.5 μg) intrastriatal infusion. While C1qa gene was locally downregulated in the ipsilateral side of injection at 12 h post-infusion, it was upregulated at the 72 h time-point. (B) Seping1 (C1-inhibitor) is upregulated in the ipsilateral side of injection by a single bolus LPS treatment. (C) Semi-quantitative analysis of Serping1 mRNA levels (differential optical density (O.D.)) in mouse brain 12 h after the intracerebral infusions. Statistical analysis was performed using student t-test, * $p < 0.05$. Abbreviations: chp, choroid plexus; m, meninges. Scale bar: as indicated in the figure.
