# Supplementary material for: Genes Involved in the Balance between Neuronal Survival and Death during Inflammation
Source: PLoS One. 2007 Mar 21;2(3):e310. doi: 10.1371/journal.pone.0000310 (PMC1819560; doi:10.1371/journal.pone.0000310)
Supplement: Figure S3 — Cellular site of transcript synthesis (0.42 MB PDF) [file pone.0000310.s005.pdf]

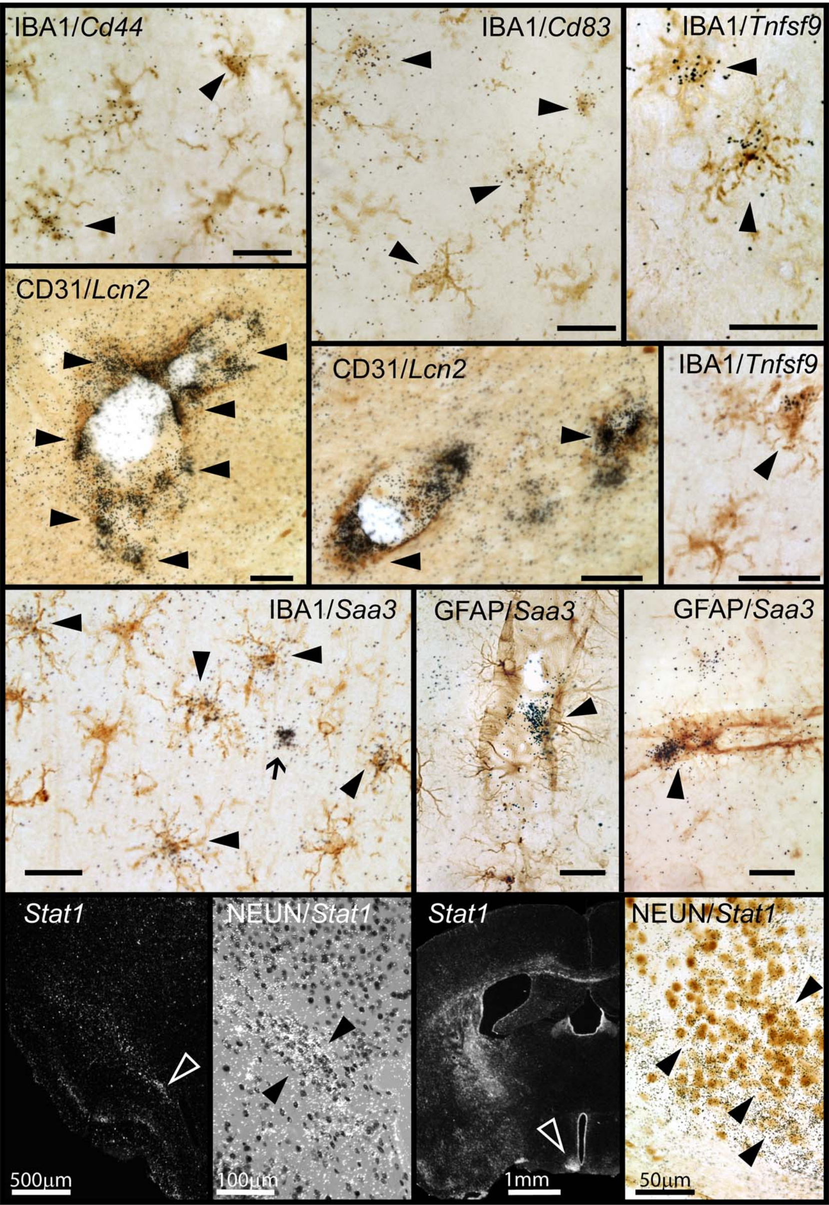
IBA1/*Cd44*
IBA1/*Cd83*
IBA1/*Tnfsf9*
CD31/*Lcn2*
CD31/*Lcn2*
IBA1/*Tnfsf9*
IBA1/*Saa3*
GFAP/*Saa3*
GFAP/*Saa3*
Stat1
NEUN/*Stat1*
Stat1
NEUN/*Stat1*
500μm
100μm
1mm
50μm

Figure S3 – Type of cells expressing the different validated genes in response to LPS or LPS/RU486 treatment. For clarity of representation in each panel, the protein and the transcript are indicated by the use of capital and italic letters, respectively. The different type of cells were identified by immunohistochemistry using peroxidase (brown ramified or elongated cells, or round nuclei), while mRNA was revealed by radioactive *in situ* hybridization (silver grains). The double-labeled cells are indicated by the black arrowheads. Silver grain accumulation not overlapping imunostained cells were pointed by the black arrows. Empty white arrowheads indicate the region detailed in high magnification in the adjacent figure at the right side. Sections were from animals sacrificed 12 h after LPS infusion. Except for Cd83 and Tnfsf9, all brain sections were from LPS single treatment, otherwise, animals received LPS/RU486 combined treatment. While Cd44, Cd83 and Tnfsf9 co-localized with IBA1-positive cells (microglia/macrophages), Saa3 co-localized with both IBA1- and GFAP-immunoreactive cells, indicating that this transcript is also synthesized in astrocytes. Stat1, differently, co-localized with NEUN-positive cells (neuronal lineage). Scale bars: 25 μm, unless indicated in the figures.
