# Supplementary material for: Genes Involved in the Balance between Neuronal Survival and Death during Inflammation
Source: PLoS One. 2007 Mar 21;2(3):e310. doi: 10.1371/journal.pone.0000310 (PMC1819560; doi:10.1371/journal.pone.0000310)
Supplement: Figure S4 — Cp expression and IBA1-Vs MAC2-positive cells (0.44 MB PDF) [file pone.0000310.s006.pdf]

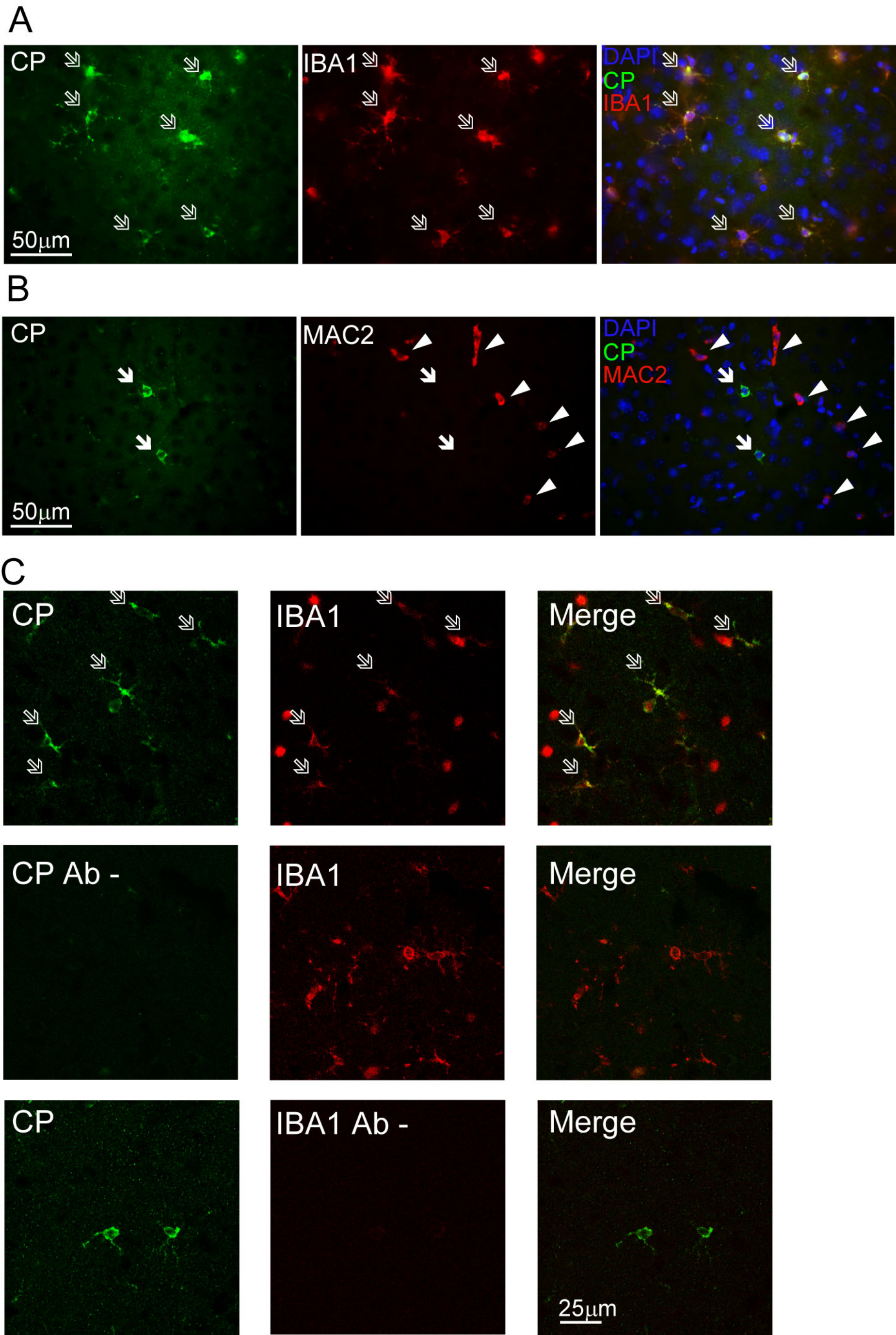
A
CP
IBA1
DAPI
CP
IBA1
50μm
B
CP
MAC2
DAPI
CP
MAC2
50μm
C
CP
IBA1
Merge
CP Ab -
IBA1
Merge
CP
IBA1 Ab -
Merge
25μm

Figure S4 – Comparative co-localization of Ceruloplasmin protein expression with IBA1- and MAC2-positive cells. (A) Epifluorescence photomicrograph depicting Cp protein (CP - green) or IBA-1 (red) immunolabeling onto brain slices of a mouse brain challenged with LPS (2.5 μg) and sacrificed 24 h post-injection. Ramified Cp protein-positive cells are comparable to IBA1-stained cells morphology/localization. IBA1-positive cells are widely distributed throughout the brain parenchyma. (B) Similar to "A" however, in this panel, red labeled cells represent MAC2-positive cells. Cells expressing MAC-2, unlike IBA1-posite cells, presented restricted distribution to the ipsilateral side of LPS injection. These cells were mostly amoeboid-like and rarely overlapped with CP-positive cells. (C) As CP and IBA1 antibodies were generated in rabbits, we performed proper controls to discard the possible artifact of co-localization between CP and IBA1. Each horizontal series of pictures represent the following combinations analyzed by confocal laser scaning: first row, both primary antibodies were used in sequential manner; second row, CP primary antibody was omitted; third row, IBA1 primary antibody was omitted. Please refer to "Supplementary Experimental Procedures" section for details. Scale bars: as indicated for each set of experiment; (A) and (B) were pictures taken at the same magnification. Empty white arrows: cells that co-localize; full white arrows: superimposed position of CP-positive cells that do not overlap; white arrowheads: MAC2-positive cells.
