# Supplementary material for: Genes Involved in the Balance between Neuronal Survival and Death during Inflammation
Source: PLoS One. 2007 Mar 21;2(3):e310. doi: 10.1371/journal.pone.0000310 (PMC1819560; doi:10.1371/journal.pone.0000310)
Supplement: Figure S5 — Time-dependent extracellular Cp expression (0.31 MB PDF) [file pone.0000310.s007.pdf]

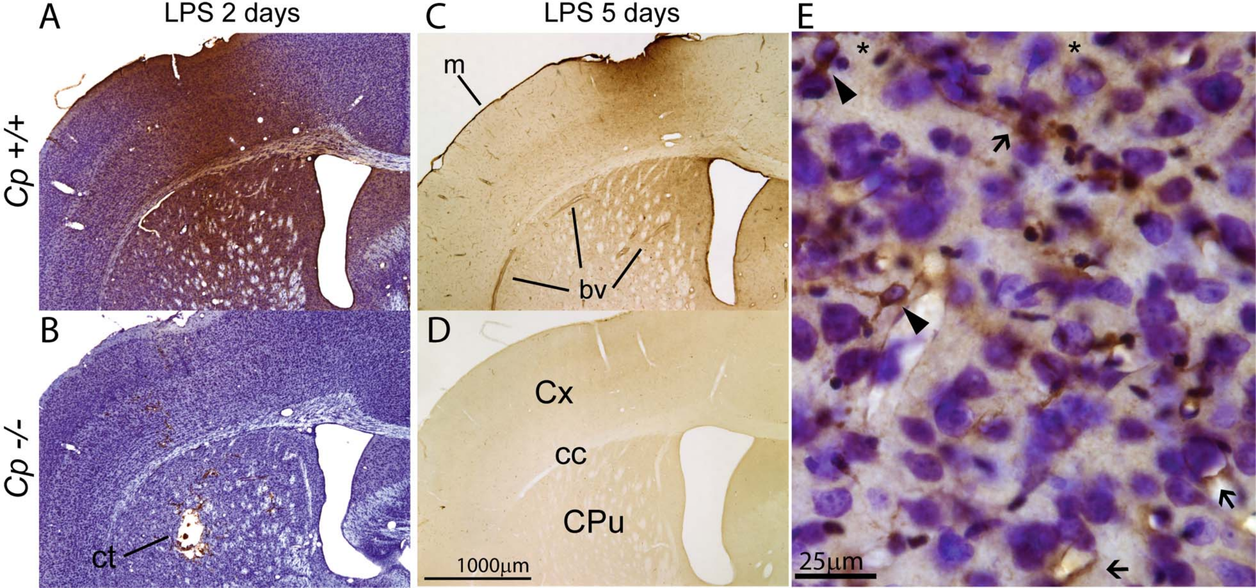

A
LPS 2 days
C
LPS 5 days
E
Cp +/+
Cp -/-
B
D
m
bv
*
Cx
cc
CPu
ct
1000µm
25µm

Figure S5 – Ceruloplasmin extracellular immunoreactivity during inflammatory challenge. (A) Photomicrograph showing dense immunostaining (brown) of Cp 2 days after LPS (2.5 μg) intrastriatal infusion (tissue was thionine counter-stained - blue) in *Cp* +/+ mice. (B) Similar treatment does not reveal any immunostaining in *Cp*-deficient mice (-/-). (C) Cp immunolabeling 5 days after LPS challenge in Cp (+/+). (D) Similar to "C", but in *Cp* (-/-) mice. (E) High magnification of staining depicted in "A". Please note the presence of Cp protein immunoreactivity at the extracellular space. Arrowheads: glial cells; Arrows, blood vessels; *, extracellular space. Abbreviations: bv, blood vessel; cc, corpus callosum; CPu, caudate putamen; Cx, cortex; m, meninges. Scale bars: as indicated in the figures.
