# Supplementary material for: Genes Involved in the Balance between Neuronal Survival and Death during Inflammation
Source: PLoS One. 2007 Mar 21;2(3):e310. doi: 10.1371/journal.pone.0000310 (PMC1819560; doi:10.1371/journal.pone.0000310)
Supplement: Text S1 — Supplementary Materials and Methods (0.18 MB PDF) [file pone.0000310.s008.pdf]

Supplementary Materials and Methods

Streptococcus Pneumoniae suspension preparation

S. Pneumoniae capsular type 6A (DBL6A) was previously described as a virulent strain (Briles et al., 1992). Hemoculture of mouse second passage isolates were provided by Dr. Denis Martin (ID Biomedical, Quebec, Canada) and a suspension of the provided culture in Todd Hewitt medium supplemented with 0.5% yeast extract (THY) was incubated at 37°C x 8% CO_2 until reaching an optical density (O.D.$_{600nm}$) of 0.4. Thereafter, 500 ml of the suspension [8 x 10^{7} colony forming units (C.F.U.)] was injected i.p. in Balb/c male mice. In the next day, mice were anesthetized for cardiac puncture and sacrifice. The blood drawn via cardiac puncture was supplemented with glycerol (20%) and frozen at -80°C. In the day of the infection, an 50 ml aliquot of the cardiac puncture was suspended in 5 ml of THY medium and incubated as described. This preparation was serially diluted until a working suspension of 50 -100 C.F.U./ml. For dose verification, 100 ml of the suspension was plated on blood-agar from two different sources (Fisher Scientific and agar plates provided by a local microbiology laboratory).

Quantitative Polymerase Chain Reaction (qPCR)

cDNA corresponding to 20 ng of the initial total RNA was used to perform fluorescence-based real-time PCR quantification using the LightCycler® Realtime PCR apparatus (Hoffman-La Roche). The FastStart DNA MasterPLUS SYBR Green Kit (Roche Diagnostics) was used as described by the manufacturer. Oligonucleotides to amplify converted Cp mRNA were: 5'-GCC CAT TGA TTG TTT GTC GGA AGT CT-3' and 5'-TCA TTG CCC ATT CCC ATC AGA TAC C-3'. The conditions for PCR were denaturation at 95°C for 10 s, annealing at 63°C for 5 s, and elongation at 72°C for 12 s followed by 79°C for 3 s (the

melting temperature of the DNA fragment). Reading of the fluorescence signal was taken at the end of the heating to avoid nonspecific signal, and a melting curve was performed to assess nonspecific signal. To avoid errors due to RNA and cDNA preparation and handling, a first correction with a housekeeping gene, subunit O of ATPase (Atp5o), was performed at each assay. The use of a standard curve allowed fit point and second derivative methods for the determination of a crossing point that was converted to express mRNA levels as copies/microgram total RNA.

Immunohistochemistry and Immunofluorescence

For Cp immunohistochemistry, free-floating sections were incubated for 30 min in KPBS containing 4% goat serum, 1% BSA, and 0.4% Triton X-100. Using the same buffer solution composition, the sections were incubated for 15 hours at 4°C with primary antibody (polyclonal rabbit anti-ceruloplasmin, 1:5000, Dakocytomation, Carpinteria, CA). After incubation with the primary antibodies, brain slices were rinsed in KPBS and incubated with a mixture of KPBS plus 0.4% Triton X-100 plus 1% BSA plus goat anti-rabbit biotinylated secondary antibody (Vector Laboratories, Burlingame, CA) for 90 min. Sections were then rinsed with KPBS and incubated at room temperature for 60 min with an avidin-biotin-peroxidase complex (Vectastain ABC elite kit; Vector Laboratories). After several rinses in KPBS, the brain slices were reacted in a mixture containing sterile KPBS, the chromogen 3,3'-diaminobenzidine tetrahydrochloride (0.05%), and 0.003% H_2O_2. Antibody specificity was verified using brain sections of *Cp* deficient mice.

For double immunofluorescence staining, tissue sections were incubated with primary antibody against Cp as described above. Tissue was washed then and incubated 120 min with fluorochrome-conjugated goat anti-rabbit Alexa-488 (Molecular Probes) at 1:500 dilution. After several washes, sections were incubated with primary antibodies against CD31, GFAP

or IBA1 as described for immunohistochemistry/*in situ* hibridization double-labelling, or with rat anti-MAC-2 (ATCC) at 1:1000 dilution. Secondary detection was performed using anti-rabbit, anti-rat or anti-mouse immunoglobulin antibodies conjugated to Cy3 (1:500, Jackson ImmunoResearch).

cRNA probes preparation

The riboprobes for mouse Cd44, Cp, Il1b-3'UTR, Selp, Plp1 and Tnfsf9 were generated from pCRII-topo vectors (Invitrogen) containing amplification PCR products of a mouse spleen (Cd44 and Cp), or brain (Plp1), or lung (Selp) cDNA libraries, or were amplified from a template plasmid containing the cDNA [Il1b (provided by Dr. P. Gray, Genentech, South San Francisco, CA) and Tnfsf9 (provided by Dr Hideo Yagita, Juntendo University School of Medicine, Tokyo, Japan)]. The list of primers and size of the products are described in table 2. All other plasmids were obtained from American Type Culture Collection (ATCC), previously described or kindly provided by other researchers, as specified in table 1. All plasmids were analyzed for sequence confirmation and orientation.

Because Cd44 is passive of multiple splicing variants, two probes for the relatively stable regions (the five first exons and the four last exons) were tested and generated similar results (data not shown in the manuscript). Since affymetrix probe set for Il1b was designed against a 3'-untranslated region (UTR), we used an Il1b probe corresponding to this same segment (preliminary tests showed that full length and 3'- UTR probes are equivalent – data not shown in the manuscript).

Table 1. Plasmids and enzymes used for the synthesis of the cRNA probes (mouse).

Plasmid for probe	Vector	Lengh (bp)	Enzymes used for the sense probe	Enzymes used for the antisense probe	Source
Saa3	pCMV-Sport6	530	NotI/Sp6	SalI/T7	American Type Culture Collection (Manassas, VA)
Lcn2	pBluescript II KS+	853	HindIII/T3	XhoI/T7	Dr Nilsen-Hamilton (Iowa State University, IA)
Serpina3n	pCMV-Sport6	~2000	NotI/Sp6	SalI/T7	American Type Culture Collection (Manassas, VA)
Gem	pCMV-Sport6	2009	NotI/Sp6	SalI/T7	American Type Culture Collection (Manassas, VA)
Cd83	pT7T3D-PacI	1561	NotI/T7	EcoRI/T3	American Type Culture Collection (Manassas, VA)
Tnfsf9	PCRII-topo	421	BamHI/T7	NotI/Sp6	Cloned by PCR (see Table 2); template cDNA provided by Dr Hideo Yagita (Juntendo University School of Medicine, Tokyo , Japan)
Cd44 (5 first exons)	PCRII-topo	462	EcoRV/Sp6	HindIII/T7	Cloned by PCR (see Table 2)
Cd44 (4 last exons)	PCRII-topo	406	HindIII/T7	EcoRV/Sp6	Cloned by PCR (see Table 2)
Stat1	pCMV-Sport6	2643	NotI/Sp6	SalI/T7	American Type Culture Collection (Manassas, VA)
Ch25h	pCMV-Sport6	1389	NotI/Sp6	SalI/T7	American Type Culture Collection (Manassas, VA)
I11b (3'-UTR)	PCRII-topo	458	BamHI/T7	EcoRV/Sp6	Cloned by PCR (see Table 2); template cDNA provided by Dr. P. Gray, Genentech, South San Francisco, CA)
C1qa	pCMV-Sport6	1080	NotI/Sp6	SalI/T7	American Type Culture Collection (Manassas, VA)
Serping1	pCMV-Sport6	~1720	NotI/Sp6	SalI/T7	American Type Culture Collection (Manassas, VA)
Hexb	pT7T3D-PacI	1008	NotI/T7	EcoRI/T3	American Type Culture Collection (Manassas, VA)
Cp	PCRII-topo	850	BamHI/T7	NotI/Sp6	Cloned by PCR (see Table 2)
Plp1	PCRII-topo	813	XbaI/Sp6	BamHI/T7	Cloned by PCR (see Table 2)
Tlr2	pCR-blunt II-topo	2278	SpeI/T7	EcoR V/Sp6	Cloned by PCR*
Nfkbia	Bluescript SK II+	1114	HindIII/T3	BamHI/T7	Dr. A. Israël (Institut Pasteur, Paris, France)
Selp	PCRII-topo	1024	BamHI/T7	EcoRV/Sp6	Cloned by PCR (see Table 2)

* Laflamme et al, 2001

Table 2. Primers used for amplification of the cDNA fragments used for riboprobe synthesis (mouse).

Plasmid	Upper Primer	Lower Primer	Reference Sequence	Lengh
Tnfsf9	5'- TGCCCCAACACTACACAACAG -3'	5'- GATAAGCCCTCAGACCCACAC -3'	NM_009404	421 bp
Cd44 (5 first exons)	5'- CCGCTACGCAGGTGTATTCC -3'	5'-CCGCTGCTGACATCGTCATCT -3'	NM_009851	462 bp
Cd44 (4 last exons)	5'-TTCCCGCACTGTGACTCAT -3'	5'- TAATGGCGTAGGGCACTACAC -3'	NM_009851	406 bp
Il1b (3'-UTR)	5'-GCAAAGTGGAGTTTGAGTCTGCA-3'	5'- GCTATGACCAATTCATCCCCCA -3'	NM_008361	458 bp
Cp	5'-TTATTTCAGTTGACACGGAACAGT-3'	5'-GGCCACCATATAAGCATCAATTA-3'	NM_007752	850 bp
Plp1	5'-GGGCTTGTTAGAGTGTTGTGC-3'	5'-CCCATGAGTTTAAGGACGG-3'	NM_011123	813 bp
Selp	5'- CTGGCTGCCCAAAAGGTTCC -3'	5'- GGTTCCTGTAGATGGGACGC -3'	NM_011347	1024 bp

Microarray Statistical Analysis

Microarray expression values were generated using RMA (Irizarry et al., 2003). Only probe sets that passed a preliminary filter were further considered for statistical analysis (Diagram A). The three-step non-specific filter was based on: signal (intensity > Log2(40) in at least 5 chips); variability ([Quantile(0.95) – Quantile(0.05)]/ mean intensity > 0.045); and probe set design (removal of s_at and x_at extension probe sets). The filtered log2-transformed expression values were fitted to a linear model according to Limma package (available at http://www.bioconductor.org) methods. A three factor model was established in order to verify the effects of LPS [present or absent (Saline)], brain side (ipsilateral or contralateral) and RU486 [present or absent (DMSO)], and relevant interactions. Statistical significance between groups of interest was assessed for relevant contrasts using moderated statistics as implemented in Limma package. We used false discovery rate corrected p-values to control false positives for the considered contrasts.

In order to determine genes significantly modulated by LPS, differentially expressed genes according to the following three contrasts (Ipsilateral/LPS/DMSO – Ipsilateral/Saline/DMSO; Contralateral/LPS/DMSO – Contralateral/Saline/DMSO; Ipsilateral/LPS/DMSO – Contralateral/Saline/DMSO) were compared in a Venn diagram (see Diagram A). After a fold change (FC) filter of at least 30% variation it was verified that all 345 probe sets that had a significant (Ipsilateral/LPS/DMSO – Ipsilateral/Saline/DMSO) contrast also presented significant (Ipsilateral/LPS/DMSO – Contralateral/Saline/DMSO) contrast; a prerequisite to consider LPS effect in ipsilateral side meaningful. No significant LPS effect was reached in the contralateral side. The 345 probe sets are plotted in centered normalized manner for illustration (Diagram A)

Selection of genes that follow RU486 main effect was performed by an alternative approach. We considered the 4 contrasts that involved RU486 – DMSO direct comparison

(i.e., Ipsilateral/LPS/RU486 – Ipsilateral/LPS/DMSO, Ipsilateral/Saline/RU486 – Ipsilateral/Saline/DMSO, Contralateral/LPS/RU486 – Contralateral/LPS/DMSO, and Contralateral/Saline/RU486 - Contralateral/Saline/DMSO). 1485 probe sets with a significant moderated F test were identified (Limma package), and were tested for correlation with a template vector designed in such way to highlight RU486 effects (Diagram A) (Pavlidis and Noble, 2001). Only genes that presented a correlation to template ≥ 0.95 and FC > 30% were selected. The Venn diagram (Diagram A) shows that there is no overlap between probe sets that follow a RU486 main effect and those with significant contrast to verify RU486 effects on LPS- induced gene expression changes (see next).

In order to screen candidate genes that are differently expressed in LPS/DMSO and LPS/RU486 conditions, a four group Venn diagram was used in order to select meaningful conditions (see diagrams B and C for illustration and details of the analysis). The four gene lists used separately in ipsilateral (Dagram B) and in contralateral (Diagram C) were: Saline/RU486 – Saline/DMSO (RU486 - Saline) contrast; LPS/RU486 – LPS/DMSO (Lps/RU486 - Lps) contrast; genes selected with a LPS significant effect (345 probe sets); LPS/RU486 – Saline/RU486 (Lps/RU486 – RU486) contrast. Please refer to diagrams B and C for details and normalized plots of probe set behaviour of the subsets.

Gene Ontology (GO) Analysis

GO biological processes nodes associated to lists of modulated genes were tested for statistical significance using a hypergeometric distribution (package GOStats http://www.bioconductor.org). In order to plot association of gene lists to biological processes in a comprehensive and comparative manner we produced a heatmap figure for GO significant nodes. In this figure GO nodes were ordered according to a hierarchical clustering procedure that considers shortest path distances between nodes. A two dimensional color

coding was used to report number of genes reported for a given node, and the corresponding significance level. The same strategy was applied for inference of molecular function and cellular components.

Complementary References

- Briles, D. E., Crain, M. J., Gray, B. M., Forman, C., and Yother, J. (1992). Strong association between capsular type and virulence for mice among human isolates of Streptococcus pneumoniae. Infect Immun 60: 111-116.
- Pavlidis, P., and Noble, W. S. (2001). Analysis of strain and regional variation in gene expression in mouse brain. Genome Biol 2: RESEARCH0042.

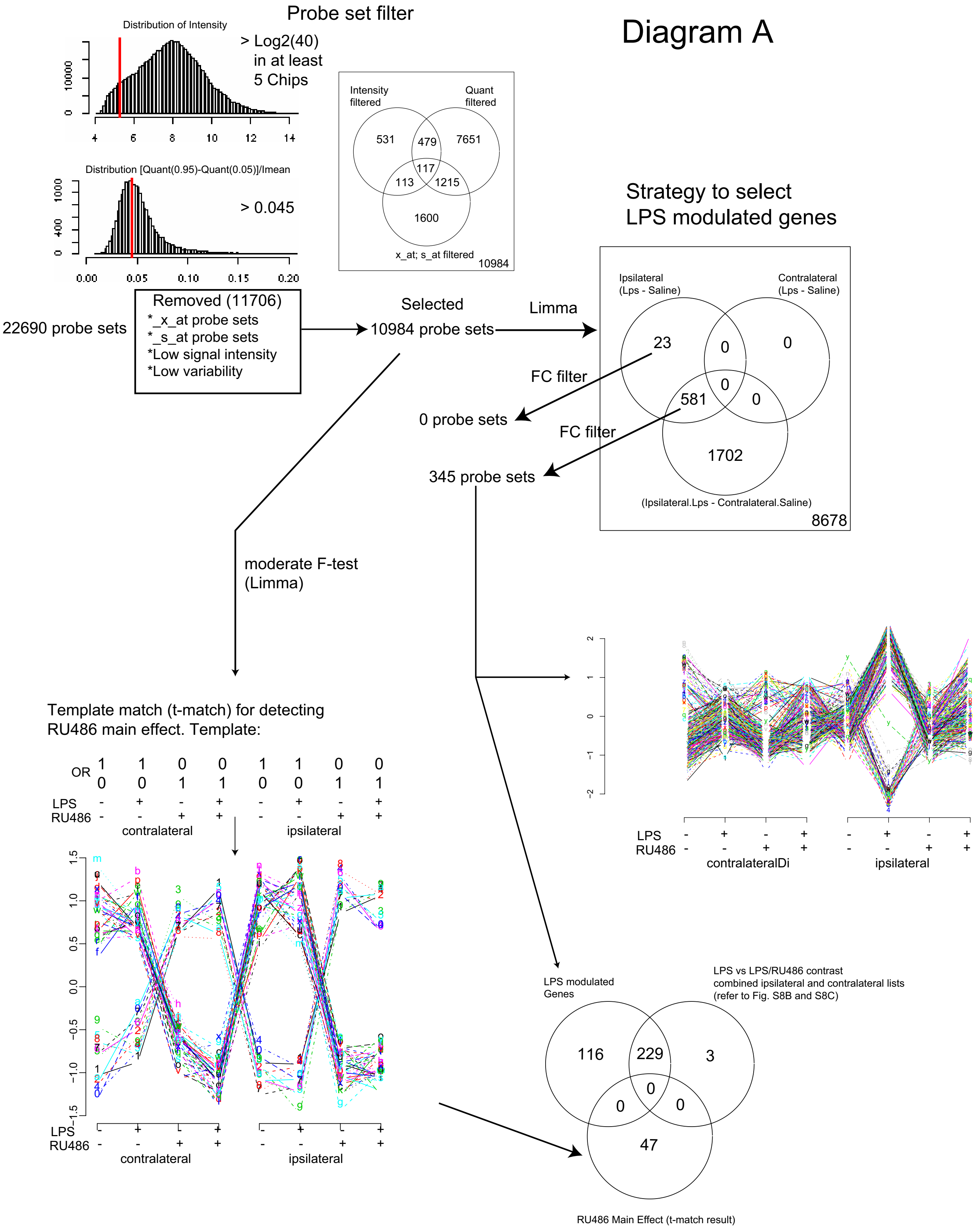
Diagram A
Probe set filter
Distribution of Intensity
> Log2(40)
in at least
5 Chips
Distribution [Quant(0.95)-Quant(0.05)]/Imean
> 0.045
Intensity filtered
Quant filtered
531
479
7651
117
113
1215
1600
x_at; s_at filtered
10984
22690 probe sets
Removed (11706)
*_x_at probe sets
*_s_at probe sets
*Low signal intensity
*Low variability
Selected
10984 probe sets
Limma
Strategy to select
LPS modulated genes
Ipsilateral
(Lps - Saline)
Contralateral
(Lps - Saline)
23
0
0
0
581
0
1702
(Ipsilateral.Lps - Contralateral.Saline)
8678
FC filter
0 probe sets
FC filter
345 probe sets
moderate F-test
(Limma)
Template match (t-match) for detecting
RU486 main effect. Template:
OR
1 1 0 0 1 1 0 0
0 0 1 1 0 0 1 1
LPS
RU486
contralateral
ipsilateral
contralateralDi
LPS modulated
Genes
LPS vs LPS/RU486 contrast
combined ipsilateral and contralateral lists
(refer to Fig. S8B and S8C)
116
229
3
0
0
0
47
RU486 Main Effect (t-match result)

Diagram B -Strategy to detect LPS vs LPS/RU486 differently expressed genes for candidate selection

Ipsilateral Side

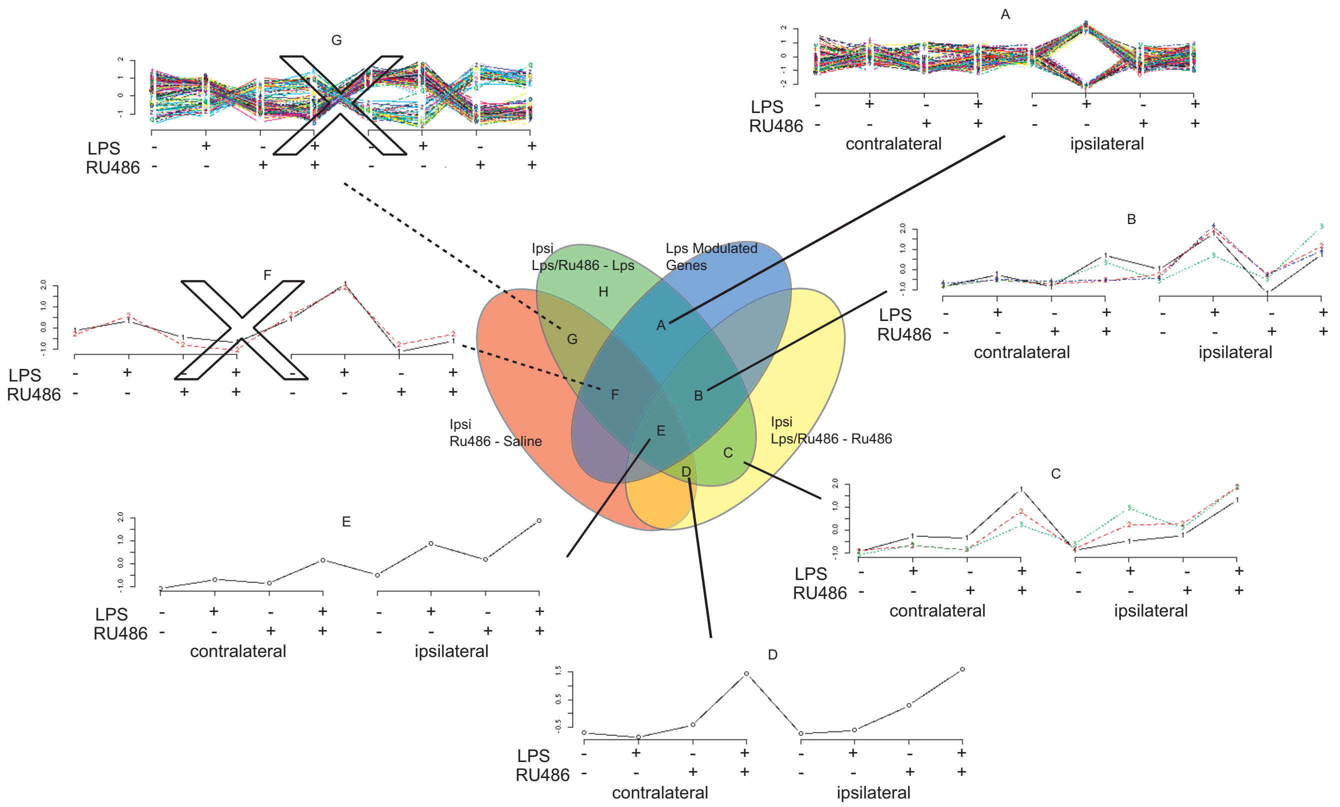

A = Lps modulated AND significative (Lps/Ru486 - Lps) contrast
B = Lps modulated AND significative (Lps/Ru486 - Lps) contrast AND significative (Lps/Ru486 - Ru486) contrast
C = significative (Lps/Ru486 - Lps) contrast AND significative (Lps/Ru486 - Ru486) contrast
D = significative (Lps/Ru486 - Lps) contrast AND significative (Lps/Ru486 - Ru486) contrast AND significative (Ru486 - Saline) contrast
E = Lps modulated AND significative (Lps/Ru486 - Lps) contrast AND significative (Lps/Ru486 - Ru486) contrast,
AND significative (Ru486 - Saline) contrast
Aftwards, Genes were filtered by Fold Change (FC) criteria: FC(Lps/Ru486 - Lps) contrast >= 30% (>=1.3 or <=1/1.3)
AND FC(Lps/Ru486 - Lps) should be at least 50% of FC(Lps - Saline):
|Log2[(FC(Lps/Ru486 - Lps)]| >= |Log2{[FC(Lps - Saline)-1]/2+1]}|

Probe sets with meaningless modulation for candidate selection AND significative (Lps/Ru486 - Lps) contrast are those in subsets "F", "G" and "H".

Diagram C -Strategy to detect LPS vs LPS/RU486 differently expressed genes for candidate selection

Contralateral Side

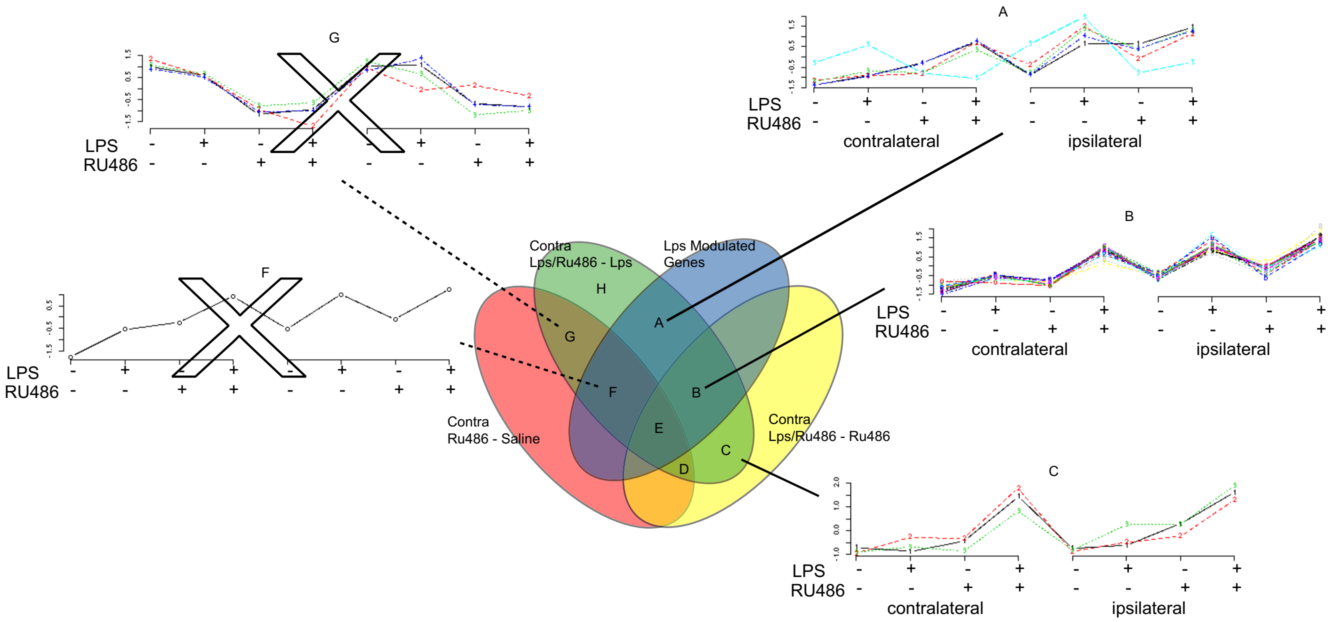

A = Lps modulated AND significative (Lps/Ru486 - Lps) contrast
B = Lps modulated AND significative (Lps/Ru486 - Lps) contrast AND significative (Lps/Ru486 - Ru486) contrast
C = significative (Lps/Ru486 - Lps) contrast AND significative (Lps/Ru486 - Ru486) contrast
D = significative (Lps/Ru486 - Lps) contrast AND significative (Lps/Ru486 - Ru486) contrast AND significative (Ru486 - Saline) contrast
E = Lps modulated AND significative (Lps/Ru486 - Lps) contrast AND significative (Lps/Ru486 - Ru486) contrast,
AND significative (Ru486 - Saline) contrast
Aftwards, Genes were filtered by Fold Change (FC) criteria: FC(Lps/Ru486 - Lps) contrast >= 30% (>=1.3 or <=1/1.3)
AND FC(Lps/Ru486 - Lps) should be at least 50% of FC(Lps - Saline):
|Log2[(FC(Lps/Ru486 - Lps)]| >= |Log2{[FC(Lps - Saline)-1]/2+1]}|

Probe sets with meaningless modulation for candidate selection AND significative (Lps/Ru486 - Lps) contrast
are those in subsets "F", "G" and "H".
